# Toll Mediated Infection Response Is Altered by Gravity and Spaceflight in *Drosophila*

**DOI:** 10.1371/journal.pone.0086485

**Published:** 2014-01-24

**Authors:** Katherine Taylor, Kurt Kleinhesselink, Michael D. George, Rachel Morgan, Tangi Smallwood, Ann S. Hammonds, Patrick M. Fuller, Perot Saelao, Jeff Alley, Allen G. Gibbs, Deborah K. Hoshizaki, Laurence von Kalm, Charles A. Fuller, Kathleen M. Beckingham, Deborah A. Kimbrell

**Affiliations:** 1 Department of Molecular and Cellular Biology, University of California Davis, Davis, California, United States of America; 2 Department of Medical Microbiology and Immunology, University of California Davis, Davis, California, United States of America; 3 Department of Biology, University of Central Florida, Orlando, Florida, United States of America; 4 Department of Genome Dynamics, Lawrence Berkeley National Laboratory, Berkeley, California, United States of America; 5 Department of Neurobiology, Physiology and Behavior, University of California Davis, Davis, California, United States of America; 6 Laverlam International, Butte, Montana, United States of America; 7 School of Life Sciences, University of Nevada, Las Vegas, Nevada, United States of America; 8 Department of Biochemistry and Cell Biology, Rice University, Houston, Texas, United States of America; Uppsala University, Sweden

## Abstract

Space travel presents unlimited opportunities for exploration and discovery, but requires better understanding of the biological consequences of long-term exposure to spaceflight. Immune function in particular is relevant for space travel. Human immune responses are weakened in space, with increased vulnerability to opportunistic infections and immune-related conditions. In addition, microorganisms can become more virulent in space, causing further challenges to health. To understand these issues better and to contribute to design of effective countermeasures, we used the Drosophila model of innate immunity to study immune responses in both hypergravity and spaceflight. Focusing on infections mediated through the conserved Toll and Imd signaling pathways, we found that hypergravity improves resistance to Toll-mediated fungal infections except in a known gravitaxis mutant of the *yuri gagarin* gene. These results led to the first spaceflight project on Drosophila immunity, in which flies that developed to adulthood in microgravity were assessed for immune responses by transcription profiling on return to Earth. Spaceflight alone altered transcription, producing activation of the heat shock stress system. Space flies subsequently infected by fungus failed to activate the Toll pathway. In contrast, bacterial infection produced normal activation of the Imd pathway. We speculate on possible linkage between functional Toll signaling and the heat shock chaperone system. Our major findings are that hypergravity and spaceflight have opposing effects, and that spaceflight produces stress-related transcriptional responses and results in a specific inability to mount a Toll-mediated infection response.

## Introduction

Human space exploration, with its promise of unprecedented discoveries, excites the imagination. However, turning the exploration of space into a practical reality presents daunting challenges including conquering the compromised biological functions produced by spaceflight. In order to achieve space exploration, a better understanding of human biology, both on earth and in space, is required. Among the many aspects of biology affected by spaceflight, we have focused on the immune response. Immune dysfunction is a major health-related problem on earth and a major obstacle to long-term space missions [1]. As early as the Apollo and Skylab missions, immune dysfunction was recognized in astronauts, and later studies documented specific host cellular and humoral immune alterations induced by spaceflight [1]. Increased microbial growth and virulence in space have also been documented [2]. Spaceflight is associated with many stresses, with altered gravitational force (g) representing the most studied factor. Microgravity (µg) is constant in space, and hypergravity (hyper g) is experienced during launch and landing. Immune dysfunction in both µg and hyper g is well documented, but determination of the underlying cellular mechanisms and thus routes to appropriate countermeasures, remains unresolved [2], [3], [4], [5], [6]. Without normal immune function, many threats to long-term survival in space exist: fatal infections, failed immunosurveillance of cancer cells, aberrant inflammatory responses and reactivation of latent viruses are all potential hazards.

In our work, we have brought advances in understanding the host defense of Drosophila to bear on deciphering the immune alterations associated with altered gravity and spaceflight. Drosophila is a well-established model for human innate immune function, sharing elements in cellular and humoral immunity, clotting and wound healing, and signaling pathways [7]. Drosophila responds to microbial infection with 1) a systemic response, characterized by fat body production of antimicrobial proteins (AMPs), 2) tissue specific responses, such as production of AMPs in the gut and trachea, 3) phagocytosis by hemocytes, and 4) clotting and wound healing [7], [8], [9], [10].

Two signaling pathways are the main mediators of the response to bacterial and fungal infections in Drosophila [7], [11], [12]. The Toll pathway primarily responds to fungal and Gram-positive (Lys-type peptidoglycan (PGN)) infections, and the Imd pathway responds to Gram-negative (DAP-type PGN) infections [7]. Toll-like receptors (Tlrs) have been identified in mammals and are the direct mediators of responses to activators such as bacterial lipopolysacccharide and viral DNA [13]. Imd shares homology with the death domain of the mammalian Receptor Interacting Protein of the Tumor Necrosis Factor Receptor pathway [7]. Downstream, through the conserved NF-kB/Rel protein transcription factors relish (Imd signaling cascade), and DIF and dorsal (Toll signaling cascade), the AMPs and ∼400 other genes are involved in response to infection [7], [14], [15]. Recognition of the complexity of the Toll and Imd pathways continues to grow, for example with identification of new regulators, interactions with the nervous system, and modification with aging [16], [17], [18], [19]. In contrast to mammals, in Drosophila only the original Toll was associated with infection response, through indirect sensing mediated by binding to Spätzle (Spz). More recently however, other Toll family members have been identified as mediating infection. Toll-8 regulates infection response in the airway epithelium [20], and Toll-7 is involved in viral recognition and response [21].

The mechanisms of interactions within and between the Toll and Imd pathways and other systems are not fully understood, and unraveling the interrelationships will require many approaches. Here, we present genetic and transcriptional profiling experiments to address the response to infection in conditions related to space travel: Does hypergravity affect the response to fungal infection? Does development during spaceflight alter the response to bacterial and fungal infections?

## Results and Discussion

### Hypergravity Increases Survival after Infection with Pathogenic Fungus

The first goal was to test our hypothesis that the immune response of Drosophila would be affected by changes in gravity at the organismal level. The simplest immune function assay is post-infection survival, and a straightforward route for altering gravity is to achieve hyper g through use of centrifuges similar to the human centrifuges used for training pilots. We infected with *B. bassiana*, an entomopathogenic fungus that enters through the cuticle and is well studied with respect to survival kinetics and Toll pathway activation [7]. Infected and control flies were then exposed to hypergravity on a centrifuge maintained at the Chronic Acceleration Research Unit (CARU), UC Davis.

**Figure 1:**
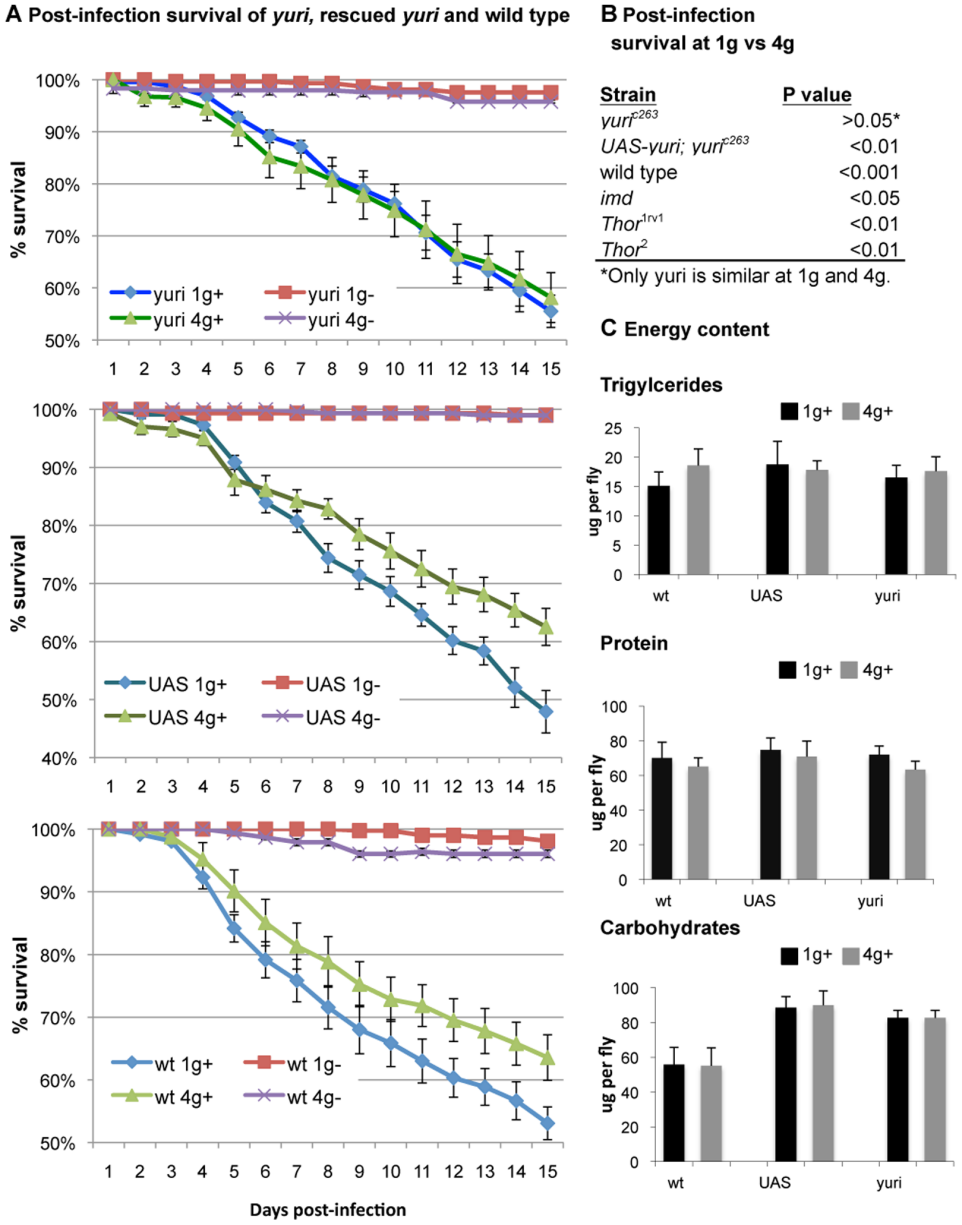
Effects of hyper g on post-infection survival and energy stores. **A.** Survival after infection with *B. bassiana* is increased by exposure to hyper g (4 g) in wild type (wt) and the rescued *yuri* strain, *yuri* ^c263^; UAS-*yuri* (UAS), but not in the gravitaxis mutant *yuri*, *yuri* ^c263^ (*yuri*). +infected, −uninfected. Error bars = SEM for 3 experiments. **B.** Additional strains tested also survive infection longer at hyper g: *imd*, using *imd^1^*, and for *Thor*, which encodes the Drosophila translational regulator 4E-BP, using *Thor^2^*, the null allele, and its control, the revertant strain Thor^1rev1^. *P* values for log rank. **C.** Post-infection energy stores of trigycerides, protein and carbohydrates are not significantly different at hyper g. Error bars = SEM for 3 experiments.

The survival of wild type and immune response mutants (except Toll pathway mutants which do not survive infection long enough for prolonged hyper g experiments) was assessed. Strikingly, all strains showed increased post-infection survival at hyper g (Figure 1A bottom panel, 1B wild type, *imd* and *Thor* strains). Given that µg is associated with impaired immune function, one interpretation of this result is that hyper g exerts the opposite effect and boosts the host response. Opposite effects of opposing gravity vectors are not uncommon, for example for platelet functions [22]. However, microorganisms can become more virulent at µg [2], and an alternative explanation is that at hyper g the fungus itself is less virulent.

In an attempt to distinguish between host and fungal responses, we tested a gravitaxis mutation of the gene *yuri gagarin* (*yuri*) [23]. *yuri* encodes 3 isoforms of a coiled-coil protein that is ubiquitously expressed, and two mutations have separate tissue specific functions related to mechanotransduction [24], [25], [26]. The *yuri^c263^* allele, caused by a GAL4 enhancer trap insertion, has defective gravity responses. A UAS-*yuri* construct, driven by the c263 transposon, rescues this phenotype through expression limited to mechanosensory neurons [23], indicating defective gravity sensing in the *yuri^c263^* mutant.

We hypothesized that if host response to hyper g were primary, then aberrant gravity sensing in *yuri^c263^* might modify the hyper g post-infection response, but if the fungal response were primary, then post-infection survival of *yuri^c263^*would be comparable to that of wild type and the immune function mutants (Figure 1AB). On testing, *yuri^c263^* failed to show this increased post-infection survival, whereas the *yuri* rescue strain had the typical increased survival response (Figure 1AB). Thus, these data demonstrate a significant host component to the hyper g effect. How might hyper g increase post-infection survival? The *yuri* finding could indicate a neural route linking mechanical load sensation to immune response. Mechanical load also affects cell biological processes [27], and one possibility is that endocytosis, which is essential for Toll signaling [28], is enhanced at hyper g. Interestingly, Yuri protein appears to have membrane-associated functions [26].

The immune response is energetically expensive, and flies with greater energetic reserves may have greater post-infection survival. However, survival did not correlate with stores of triglycerides, carbohydrates or protein (Figure 1C).

### The Fungus, Immunity, Tumorigenesis (FIT) Microgravity Experiment

These results showing that the immune response of Drosophila responds to g force formed the basis for the space shuttle experiment Fungus, Immunity and Tumorigenesis (FIT). FIT is the first flight experiment to investigate µg effects on Drosophila immunity. The FIT experiment was flown on the shuttle Discovery (STS-121), and involved an experimental design adjusted for shuttle conditions. Ideally the design would have paralleled the hyper g work, with infection of Drosophila genotypes proceeding in space. But due to flight constraints, space infections were not possible and only a single genotype could be flown. However, the flight duration (12 days) allowed production and return to Earth of a small population of flies that had undergone their entire development in space (space flies). Upon return this population was divided into three groups and used for transcription profiling without infection and after infection with *B. bassiana* or *E. coli*. The fungal spores and *E. coli* used were grown on Earth. Earth-reared flies, grown at Kennedy Space Center, were used as controls (Earth flies). Recordings relayed from the shuttle ensured similar growth conditions for the space and Earth flies other than the change in g force. The experiments thus encompass humoral immunity in response to Toll and Imd mediated fungal and bacterial infections through transcriptional profiling after development in space. The uninfected space flies showed an altered transcriptional profile, and those changes will be presented last, in the context of the immune response data.

### The Toll Pathway is Dysfunctional in Adults Raised in Space

**Figure 2:**
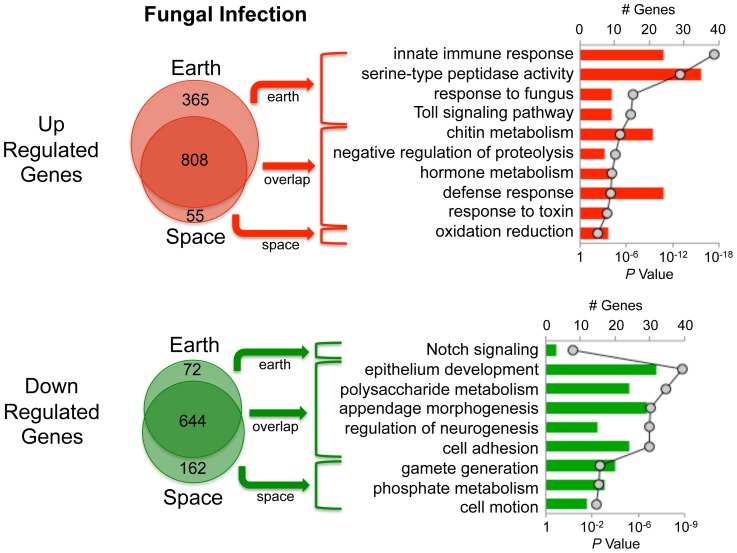
Microarray-based analysis of response to *B. bassiana*. The total number of genes upregulated or downregulated in Earth flies only (Earth) or space flies only (Space) or in both (overlap) are indicated by Venn diagrams. Pathway analysis of each of these groups is shown on the right side of the figure. The number of genes in each functional category is depicted in bar graphs (primary y-axis), and the *P* values corresponding to statistical over-representation of each category are presented as a line graph (secondary y-axis). Note that certain genes annotated into more than one of these categories.

Transcriptional profiling of space and Earth flies infected with *B. bassiana* revealed that the space flies have a dramatically different response (Figure 2). For Earth flies, the upregulated genes revealed the expected [7], [14], [15] response categories: transcripts for genes associated with innate immune response, serine peptidase activity, response to fungus and Toll signaling pathway activation were all statistically over-represented (Figure 2). In stark contrast, none of these gene categories was upregulated in space flies (Figure 2).

**Figure 3:**
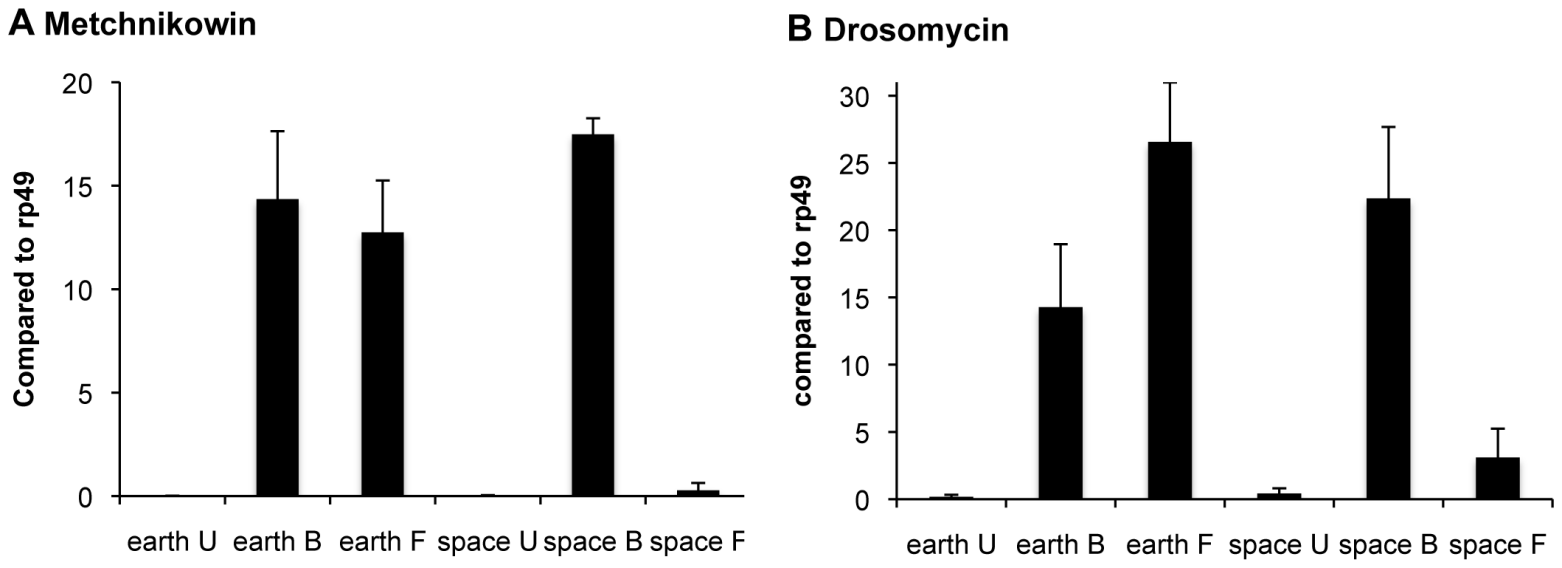
Antifungal AMPs. **A.** *Metchnikowin* and **B.** *Drosomycin* transcript levels were assessed by qPCR in space and Earth flies infected with fungus (F) or bacteria (B), or uninfected (U), and standardized by comparison to the level of ribosomal protein gene *rp49*. Error bars = SEM for 3 experiments.

**Figure 4:**
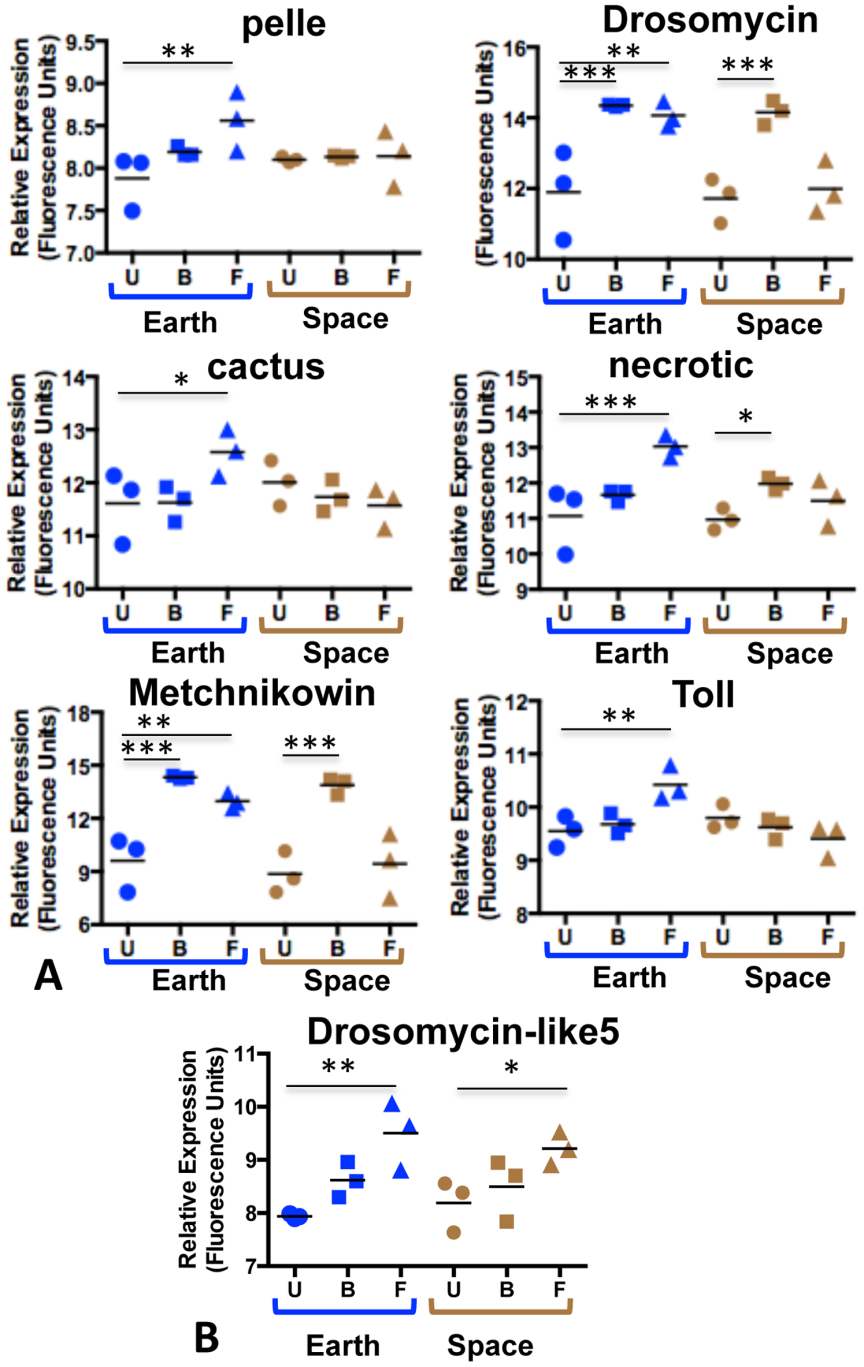
Transcriptional profiling of genes associated with the Toll pathway. **A.**Relative expression levels of selected Toll associated genes as detected by microarray are shown in uninfected (U, circles) Earth (blue) and space (tan) flies, and following fungal (F, triangles) or bacterial (B, squares) infection of space and Earth flies. Transcriptional regulation **A.** not shared or **B.** shared by space and earth flies infected with fungus.

The AMPs *Metchnikowin* and *Drosomycin* are key indices of the Toll signaling response [7]. Figures 3 and 4A present transcriptional analysis by quantitative real-time PCR (qPCR) and microarray-based analysis establishing the failed induction of these two genes in space flies. Results of microarray-based analysis for additional genes are also presented in Figure 4A. Note that *necrotic* (*nec*) is upregulated in the Earth flies, which is an indicator of a strong anti-fungal response since Nec downregulates the immune reaction via negative regulation of Persephone (Psh) [29]. A complete listing of the fold changes and associated *p* values for all the transcriptionally modulated genes of the categories shown in Figure 2 is presented in Table S1.

Collectively, these data indicate that Toll mediated responses to *B. bassiana* are impaired in space flies, and in particular the failure of *Drosomycin* and *Metchnikowin* activation indicates that the space flies are severely immunocompromised. The data do not, however, reflect a complete failure of the space flies to react to the infection. Some defense response category genes were activated in space flies as well as Earth flies (Figure 2 and Table S1), and these indicate that signaling pathways other than Toll are functional in space flies. Some of these genes are *Turandot* (*Tot*) family members, a set of genes induced under a variety of stresses such as septic infection, paraquat feeding, UV exposure and heat shock, and with complex regulation involving the Jak-Stat, Imd and Mekk1 pathways [30], [31], [32]. Also induced in both space and Earth flies are the fungal infection response genes *Thioester containing protein IV* (*Tep IV*), which has an alpha-macroglobulin complement component, and *Transferrin 1* (*Tsf1*), which is predicted to be involved in iron homeostasis. Both of these genes are also induced in response to DNA damage in the larval epidermis, as is *Tot C* [33], [34].

The only AMP gene induced in space flies by the fungal infection is *Drosomycin-like 5* (*Drsl5*) (Figure 4B). *Drsl5* induction in response to *B. bassiana* is regulated by both the Toll and Imd pathways [7]. Thus induction of *Drsl5* in the space flies is not necessarily evidence for a functional Toll response and may represent activation by the Imd pathway (see below) or another route. Both space and Earth flies upregulated genes associated with response to toxins, including cytochrome P450s (*Cyp4ac1*, *Cyp4ac2*, *Cyp4aa1*, *Cyp304a1*), which are associated with detoxification of xenobiotics and hormone metabolism [35], [36] (Table S1).

Genes induced uniquely in the space flies by fungal infection could indicate an altered infection response. However, only one category of genes emerged from microarray analysis as specifically induced by infection in space flies: oxidation/reduction (Figure 2 and Table S1). Six of the eight genes in this category are *Pyrroline 5-carboyxlate reductase* (*P5cr*), probable cytochrome P450s (*Cyp6t1*, *Cyp6t1* and *Cyp6a13*), *CG6012* and *CG10131*. The remaining two genes, *phenoloxidase subunit A3* (*PO45*) and *prophenol oxidase A1* (*proPO-A1*), have roles in melanization, which is also used as a defense against pathogens and in wound response [37]. However, other genes in the melanization cascade were upregulated in Earth flies but unchanged in the space flies, e.g. *MP1*, S*pn27A* and *Hayan* [38], [39]. In contrast, *Gram-negative binding protein 3* (*GNBP3*) is upregulated in space flies, and GNBP3 assembles defense complexes, including phenol oxidases, in a Toll independent manner [40].

Initial detection of *B. bassiana* infection occurs through dual signaling arms upstream of Spz, the only known ligand for Toll [16], [41]. In one arm, Psh, moderated by suppression from Nec, senses fungal virulence factors and other danger signals, leading to activation of the Spätzle Processing Enzyme (SPE), cleavage of the Spz prodomain, and binding of processed Spz to the Toll transmembrane receptor [29]. In the other signaling arm, GNBP3 binds fungal cell wall components and initiates a cascade via ModSP and Grass that also leads to SPE activation and Spätzle binding to Toll [16], [42]. Thus, if space flies are defective in initial sensing of the infection, a minimum of two defects are needed to block both arms of the upstream signaling cascade. If the space flies are not defective in sensing, a single non-functional step at the level of SPE or further downstream in the Toll pathway could prevent the activation of target genes.

### The Imd Pathway is Activated Normally in Adults Raised in Space

**Figure 5:**
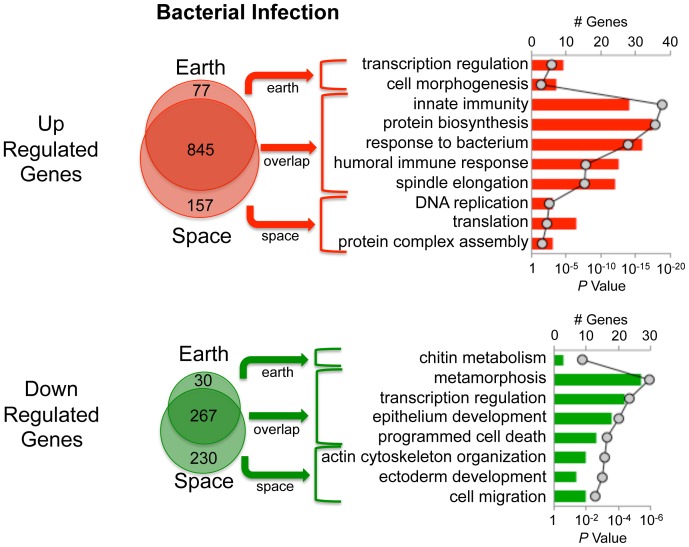
Microarray-based analysis of response to *E. coli*. The total number of genes upregulated or downregulated in Earth flies only (Earth) or space flies only (space) or in both (overlap) are indicated by Venn diagrams. Pathway analysis is shown on the right side of the figure. The number of genes in each functional category is depicted in bar graphs (primary y-axis), and the *P* values corresponding to statistical over-representation of each category are presented as a line graph (secondary y-axis).

In complete contrast to fungal infection, space flies infected with *E. coli* show strong gene expression responses similar in character to those of Earth flies (Figure 5). For both Earth and space flies, expected categories of upregulated genes [7], [14], [15] were statistically over-represented: innate immunity, response to bacterium and humoral immune response (Figure 5). Accordingly, the Imd pathway appears to have been activated normally in the space flies. Table 1 presents a subset of these genes categorized into AMPs, Peptidoglycan recognition proteins (PGRPs), Turandot, Immune induced molecules, Thioester-containing proteins, and Miscellaneous. Table S2 details all of the transcriptionally upregulated and downregulated genes in the categories presented in Figure 5. D*rosomycin* and *Metchnikowin* are included among the standard AMP genes activated by *E. coli* infection (Table 1, Figures 3 and 4). Despite its activation by a Gram-negative organism, D*rosomycin* is mainly considered a readout for Toll signaling through cross recognition extracellularly or cross talk intracellularly with Imd or another pathway [16]. As discussed below, our hypotheses on the effects of µg on Toll function would suggest the cross-reaction is downstream of Toll receptor activation. Metchnikowin has both antibacterial and antifungal activity, and can be activated transcriptionally through Imd or Toll, depending on the type of infection [43]. Thus the normal induction of *Metchnikowin* in the bacteria infected space flies, but lack of induction in the fungus infected space flies, characterizes the normal Imd signaling versus the abnormal Toll signaling of the space flies.

**Table 1:** Antibacterial response is similar in space and earth flies.

Category	Symbol	Fold changeearth	P value earth	Fold changespace	P value space
**Antimicrobial proteins**					
Defensin	Def	186.4	0.00	156.1	0.00
Drosocin	Dro	68.3	0.00	77.8	0.00
Attacin-A	AttA	82.2	0.00	68.4	0.00
Attacin-C	AttC	14.9	0.00	15.7	0.00
Attacin-D	AttD	154.1	0.00	99.5	0.00
Diptericin B	DptB	26.6	0.00	40.1	0.00
Cecropin B	CecB	4.9	0.00	5.4	0.00
Cecropin C	CecC	24.2	0.00	30.9	0.00
Metchnikowin	Mtk	26.2	0.00	32.4	0.00
Drosomycin	Drs	5.5	0.00	5.4	0.00
**Peptidoglycan recognition proteins**					
Peptidoglycan recognition protein LC	PGRP-LC	2.4	0.00	2.0	0.00
Peptidoglycan-recognition protein SC2	PGRP-SC2	15.3	0.00	8.9	0.00
Peptidoglycan recognition protein LB	PGRP-LB	8.6	0.00	8.9	0.00
Peptidoglycan recognition protein LA	PGRP-LA	2.1	0.01	2.2	0.01
Peptidoglycan recognition protein LF	PGRP-LF	5.6	0.00	3.8	0.00
Peptidoglycan-recognition protein SB1	PGRP-SB1	10.0	0.00	13.1	0.00
**Stress inducible Turandot**					
Turandot M	TotM	20.6	0.00	27.9	0.00
Turandot A	TotA	5.5	0.02	6.8	0.01
Turandot X	TotX	2.2	0.21*	3.8	0.04
Turandot C	TotC	28.0	0.00	58.2	0.00
**Immune induced molecules**					
Immune induced molecule 10	CG33470	3.7	0.00	2.2	0.04
Immune induced molecule 23	IM23	3.8	0.01	3.1	0.02
Immune induced molecule 1	IM1	3.4	0.00	3.1	0.00
Immune induced molecule 2	IM2	2.0	0.00	1.8	0.01
Immune induced molecule 4	IM4	3.1	0.01	2.9	0.01
Immune induced molecule 10	IM10	3.5	0.00	2.8	0.01
**Thiolester containing proteins**					
Thiolester containing protein I	TepI	22.2	0.00	28.4	0.00
Thiolester containing protein II	TepII	5.2	0.00	4.0	0.00
Thiolester containing protein IV	TepIV	2.7	0.00	2.9	0.00
**Miscellaneous**					
insulin-stimulated eIF-4E binding protein	Thor	2.5	0.02	2.2	0.04
eiger	egr	2.4	0.05	1.6	0.28*
Relish	Rel	3.8	0.00	2.2	0.02
Sterile alpha & TIR motif-containing protein 1	Ect4	1.7	0.06*	2.0	0.02
Inhibitor of apoptosis 2	Iap2	1.8	0.02	1.9	0.01
Hemolectin	Hml	3.3	0.00	2.6	0.00

* P value not significant, >0.05.

### Adults Raised during Spaceflight have an Altered Transcriptional Profile

Comparing the transcription profiles of uninfected space and Earth flies provides valuable insights into the biological processes affected by the µg environment and thus generates clues as to the origin of the differing effects on Imd and Toll mediated responses. These comparisons may also provide evidence relevant to the enhanced post-infection survival at hyper g.

**Figure 6:**
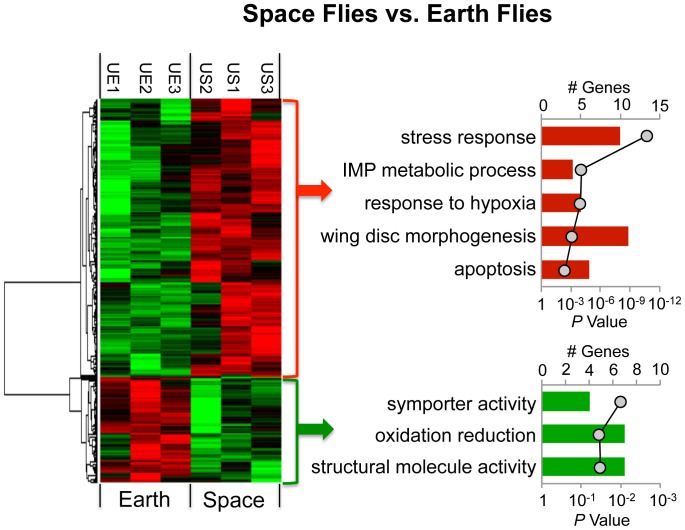
Analysis of transcriptional modulations produced by spaceflight. Transcriptional profiles of uninfected space and Earth flies were compared and differentially expressed genes were grouped by hierarchical clustering. Pathway analysis was utilized to identify statistically enriched biological themes. The number of genes in each category is depicted in bar graphs (primary y-axis), and the *P* values corresponding to statistical over-representation of each category are presented as a line graph (secondary y-axis).

The transcriptional profiles of uninfected space and Earth flies were compared by first hierarchically clustering genes that were differentially expressed between the two groups (Figure 6). Pathway analysis indicated the transcripts expressed at higher levels in space flies included a statistical over-representation of genes linked to stress response, inosine monophosphate (IMP) metabolism, response to hypoxia, wing disc morphogenesis, and apoptosis. A much smaller list of transcripts was expressed at lower levels in space flies than Earth flies, and appeared to be enriched in genes associated with symporter activity, oxidation/reduction, and structural molecule activity (Figure 6). Several immune response genes were also differentially expressed, but were not statistically over-represented as a functional category: *relish* (2.4 fold), *spätzle* (1.7 fold), *dorsal* (1.6 fold), *virus-induced RNA 1*(2.1 fold), *Serpin 28Db* (1.6 fold), serine peptidase CG18563 (2.0 fold), *pirk* (1.6 fold), and *PGRP-LF* (1.6 fold). In total, less than only 280 genes showed a significant difference in expression between space and Earth flies (Table 2, Table S3). Interestingly, 127 of these genes are uncharacterized and only identified as CG numbers [44], and may also be of interest in the spaceflight and immune context as more information is acquired.

**Table 2:** Transcriptional response of space flies without an infection.

Catagories of genes with altered response					
	Symbol	Fold Change		Symbol	Fold Change
**Stress Response (P value 1.59E-11)**			**Response to Hypoxia (P value 1.07E-04)**		
Heat shock protein 70Bc	Hsp70Bc	13.0	Heat shock protein 70Bc	Hsp70Bc	13.0
Heat shock protein 70Aa,Ab	Hsp70Aa,Ab	9.1	hairy	h	1.9
Heat shock protein 22	Hsp22	5.4	Heat shock protein 70Aa,Ab	Hsp70Aa,Ab	9.1
Heat shock gene 67Bc	Hsp67Bc	3.4	Heat shock protein 70Ba	Hsp70Ba	11.4
Heat shock protein 70Ba	Hsp70Ba	11.4	Heat shock protein 23	Hsp23	2.8
Heat shock protein 26	Hsp26	3.3	**IMP Metabolic Process (P value 8.24E-05)**		
Heat shock protein 23	Hsp23	2.8	adenosine 3	ade3	1.6
Heat shock protein 27	Hsp27	3.6	Dmel_CG11089	CG11089	1.6
Heat shock gene 67Ba	Hsp67Ba	1.5	Phosphoribosylamidotransferase 2	Prat2	1.6
Heat shock protein 68	Hsp68	5.0	adenosine 2	ade2	2.0
**Wing Disc Morphogenesis (P value 7.88E-04)**			**Apoptosis (P value 3.86E-03)**		
tolkin	tok	1.5	Protein kinase 61C	Pdk1	1.7
pangolin	pan	1.9	Rep3	Drep-3	1.5
Tissue inhibitor of metalloproteases	Timp	1.5	Strica	dream	1.6
Star	S	1.6	MAP kinase-activating death domain protein	rab3-GEF	1.5
LIM-kinase1	LIMK1	1.5	starvin	stv	2.1
knot	kn	1.5	Death associated molecule related to Mch2	Damm	2.1
schnurri	shn	1.5	**Symporter Activity (P value 9.82E-03)**		
lethal (2) giant discs 1	l(2)gd1	1.6	Dmel_CG8083	CG8083	−2.2
downstream of receptor kinase	drk	1.5	Dmel_CG9826	CG9826	−2.2
Suppressor of cytokine signaling at 36E	Socs36E	2.3	Sodium-dependent multivitamin transporter	Smvt	−1.9
rhomboid	rho	1.9	lethal (2) 01810	dmGlut	−1.6
**Oxidation Reduction (P value 3.50E-02)**			**Structural Molecule Activity (P value 3.18E-02)**		
Dmel_CG3609	CG3609	−1.5	obstructor-B	obst-B	−1.6
Ribonucleoside diphosphate reductase large subunit	RnrL	−1.5	mitochondrial ribosomal protein S28	mRpS28	−1.7
lysyl oxidase-like 2	lox2	−1.6	Cuticular protein 92A	Cpr92A	−1.9
Dmel_CG15629	CG15629	−1.5	mitochondrial ribosomal protein L21	mRpL21	−1.5
prolyl-4-hydroxylase-alpha SG2	PH4alphaSG2	−1.5	Yolk protein 2	Yp2	−2.0
Photoreceptor dehydrogenase	Pdh	−1.5	Cuticular protein 62Bb	Cpr62Bb	−1.6
Dmel_CG12539	CG12539	−1.5			

Current annotations show that the most striking alterations are in expression of heat shock protein genes, a subset in the stress response category (Table 2). The heat shock response is evolutionarily conserved and perhaps the most well studied stress response [45]. Heat shock proteins also function under normal conditions, and in general act as molecular chaperones assisting in forming, or regaining, the normal folding of polypeptides, translocating proteins, and regulating protein degradation [45], [46]. The heat shock response occurs in reaction to many types of stress and is usually initiated by unfolded/misfolded proteins. In correcting this cytotoxic state heat shock proteins also inhibit apoptosis [45], [47]. Given their functions, it is not surprising that heat shock gene expression changes have been associated with altered gravity and spaceflight in a variety of organisms; however, results are variable and a clear picture of heat shock protein involvement in these situations has not emerged [48], [49], [50], [51].

Two further categories of altered gene expression are noteworthy with respect to the heat shock result seen for the space flies: apoptosis and response to hypoxia (Table 2). Six genes associated with apoptosis are upregulated: *starvin*, which is a cochaperone associated with heat shock protein 70 (Hsp70) [52]; the caspase *Damm*, which can trigger apoptosis when overexpressed [53]; *Pdk1*, a serine/threonine kinase that is a negative regulator of apoptosis [54]; *Drep-3*, one of four Drosophila DNA fragmentation factor-related proteins [55]; *dream*, a serine threonine rich caspase [56]; and *Rab3-GEF*, a Ras superfamily member predicted to regulate the cell cycle and apoptosis [57]. The response to hypoxia category includes a subset of the heat shock protein genes and *hairy*, a master regulator for adjustment to hypoxia [58]. Together these transcriptional alterations indicate severe stress associated with protein unfolding during development of the flies in µg.

Do these changes in the space flies provide insight into the failed immune response to fungal infection versus the robust immune response to bacterial infection? Although differences in the physiologies of the two infections, i.e. acute infection by the non-pathogenic *E.coli* and chronic infection by the pathogenic *B. bassiana*, may play some role here, the strong heat shock response produced by the space environment offers two testable molecular hypotheses.

#### Hypothesis 1

The extracellular space is more susceptible to protein unfolding in stress conditions than the intracellular environment. Thus in the µg conditions experienced by the space flies, the more complex extracellular induction events associated with Toll activation (recognition, activation of SPE, cleavage of Spz and binding to Toll) are more susceptible to disruption than those associated with activation of the Imd pathway. For the Imd pathway, the extracellular event is direct binding of bacterial components, PGN, to cell surface receptors, PGRPs [19]. A corollary of this hypothesis is that, in time, the heat shock proteins may mediate recovery of Toll signaling.

#### Hypothesis 2

Heat shock protein(s) interferes directly with the binding of (processed) Spz to Toll. In mammals, extracellular heat shock proteins bind directly to Tlr receptors and are important in moderating the immune response, including in the clinical setting [59], [60], [61]. In contrast in Drosophila, Spz is the only known ligand for Toll [16], [42]. Heat shock proteins do not, however, need to be Toll ligands in order to interfere with Spätzle binding, or to inhibit activity of essential upstream components such as SPE, Psh and Grass. A corollary is that heat shock proteins may be both positive and negative regulators of the Toll signaling pathway, inhibiting or enhancing according to the conditions. This corollary is analogous to the positive and negative regulation of Tlrs effected by extracellular heat shock proteins in mammals [62].

These hypotheses on heat shock protein mediation of the effects of g force on immune responses have broad implications, providing insights into established findings, suggestions for further experimentation and predictions for other stressful conditions. One clear, testable, inference is that the compromised human immunity seen at altered g results from protein unfolding and heat shock protein engagement. Our hypotheses also suggest an underlying mechanism for our hyper g findings. Thus hyper g may stabilize proteins against unfolding or affect heat shock protein interaction with Toll receptors. Effects on the stability, folded status or function of endocytotic components may be particularly important both at hyper g and µg since endocytosis is essential for Toll, but not Imd, signaling [28]. A further possibility is that most common stresses such as sleep deprivation, physical activity, and ageing, affect immune responses via these proposed routes.

Other studies have noted the opposing effects of increased and reduced g force on expression of individual Drosophila genes in uninfected flies [50], [63]. In addition, in one µg experiment, phagocytosis in adult Drosophila females, but not larvae, raised in space was reported to be normal, and expression of a few antimicrobial genes was altered in these adults by infection with an *E. coli* strain that does not grow in Drosophila [64]. In the future, experiments on board the International Space Station (ISS), where multi-generational studies with multiple strains of flies and pathogens are possible, would provide an optimal route for testing the hypotheses suggested here. Other factors that might affect microgravity immune responses - such as the route for pathogen delivery, developmental events, microbiome, and signaling pathway modulation by epigenetics or non-coding RNA activity - could also be addressed. The key to applying the full capacity of Drosophila aboard the ISS for an understanding of gravitational effects on innate immunity will be the use of a wide range of pathogens, genotypes, and approaches by many different investigators.

The juxtaposition of our µg and hyper g findings highlights the importance of gravity in normal immune function and begins to elaborate the key cellular and molecular components of the immune system that respond to changes in gravity. Our findings also suggest that exposure to gravity may mitigate the deleterious physiological, including immune, consequences of spaceflight and provide a rationale for including human centrifuges on facilities for long-term transport and housing of humans in space.

## Materials and Methods

### Drosophila Stocks

All experiments used only males. Oregon-R wild-type flies were used. Others are: *imd^1^* (Flybase FB, FBal0045906), *yuri* ^c263^ and UAS-*yuri* (FBgn0045842 and [23]), Thor^2^ and Thor^1rev1^ (FBgn0261560 and [65], [66]), *spz^4^* (FBal0016062) and *imd^EY08573^* (FBal0159146) [44]. The stock for the space and Earth flies, hemolectin-Gal4; UAS-GFP, expressed GFP in the blood cells [44]. The space containers are presented in Marcu et al. [64].

### Microorganisms and Infections

Bacterial infections with *E. coli* ATCC 25922 were as previously described [65], [67]. A single spore isolate of *Beauveria bassiana* (strain GHA) was cultured on Sabouraud dextrose agar. Conidia and hyphae were harvested by passing culture through a sterile ASTM No. 100 sieve. Spores were also flown on the space shuttle and we are happy to provide information upon request. Natural infection by *B. bassiana* used a dosage of 9.5×10^6^ spores/fly, with procedures and survival assays as previously described [67]. Ten replicates of 20 flies each for all strains were used in all 3 experiments for the CARU hypergravity tests. The centrifuge was stopped once per day to conduct survival counts. Control survival assays after bacterial and fungal infections on wild-type, hemolectin-Gal4; UAS-GFP, *imd^1^*, *imd^EY08573^* and *spz^4^* [67] were conducted at Kennedy Space Center to establish that space and Earth fly infections were proceeding in accordance with our standardized conditions.

### Energy Content

Flies were homogenized in a solution containing 1% NP-40, 0.5% deoxycholic acid, 0.1% Triton-X 100, 100 mM NaCl, 0.1 mM CaCl_2_, and 2 mM MgCl_2_, pH 7.6. Homogenates were heated for 5 min at 75°C to inactivate lipases. Triacylglyceride levels were measured using a commercial serum triglyceride kit (Sigma; St. Louis, Missouri USA; No. TR0100-1KT), and protein content was quantified using the bicinchoninic acid method [68]. Carbohydrates (glycogen and trehalose) were digested with amyloglucosidase and quantified with a blood glucose kit (Pointe Scientific; Canton, Michigan, USA; No. G7521). 4–14 flies were assayed for each treatment group, and all assays were performed in triplicate.

### Gene Expression Analysis

Total RNA was extracted from flies utilizing the Qiagen RNeasy© RNA isolation kit. mRNA amplification, labeling, hybridization to Drosophila Genome 2.0 GeneChips© (Affymetrix), staining and scanning were performed as previously described [69] utilizing protocols in the Affymetrix Gene Expression Analysis Technical Manual. RMA-based, (Partek Genomics Suite^©^, v.6.6) algorithms were used to identify differentially expressed genes (DEG). Three replicate samples were included in each control and experimental group. A minimum fold-difference of +/−1.5 (*p*-value ≤0.05) was used as the cut off criteria for generating DEG lists. DEG lists were hierarchically clustered and sub-clusters were subjected to pathway analysis using Ingenuity Pathway Analysis (IPA^©^) and DAVID (http://apps1.niaid.nih.gov/david) web-interfaced software. GEO accession number is GSE53196. qPCR analysis was carried out on cDNA made using the Bio-Rad iScript™ cDNA Synthesis Kit (No. 170–8891) and utilized the Bio-Rad SsoAdvanced™ SYBR® Green Supermix (No. 172–5260). Reaction mixtures were prepared as specified in the product protocol and used QuantiTect Primer Assays from Qiagen: Metchnikowin (No. QT01109619), Drosomycin (No. QT00957432), and RPL32 (rp49) (No. QT00985677). Samples were run in triplicate on the Bio-Rad qPCR CFX thermal cycler.

## Supporting Information

Table S1 Individual genes for all categories of the response to *B. bassiana* in Figure 2.
(PDF)

Table S2 Individual genes for all categories of the response to *E. coli* in Figure 5.
(PDF)

Table S3 Genes with altered response in uninfected space flies, in addition to those in Table 2.
(PDF)
