# Supplementary material for: Toll Mediated Infection Response Is Altered by Gravity and Spaceflight in Drosophila
Source: PLoS One. 2014 Jan 24;9(1):e86485. doi: 10.1371/journal.pone.0086485 (PMC3901686; doi:10.1371/journal.pone.0086485)
Supplement: Table S1 — Individual genes for all categories of the response to B. bassiana in Figure 2. (PDF) [file pone.0086485.s001.pdf]

Table S1. Individual genes (Title and Symbol) for all categories of the response to B. bassiana in Figure 2. Fold change in earth flies (EF), space flies (SF), and corresponding P values (P).

Innate Immune Response (IEF only)

**Title**	**Symbol**	**EF**	**P**	**SF**	**P**
Serine Protease Immune Response Integrator	spirit	3.1	0.00	1.2	0.31
Peptidoglycan recognition protein SA	PGRP-SA	3.0	0.00	1.3	0.18
cactus	cact	2.0	0.02	-1.3	0.26
fondue	fon	1.6	0.02	-1.0	0.80
Peptidoglycan-recognition protein SC2	PGRP-SC2	3.5	0.00	1.3	0.42
Attacin-C	AttC	2.4	0.04	-1.3	0.46
Immune induced molecule 10	CG33470	6.3	0.00	1.6	0.18
Attacin-A	AttA /// AttB	10.0	0.00	1.6	0.45
Metchnikowin	Mtk	10.3	0.00	1.5	0.55
Immune induced molecule 23	IM23	4.7	0.00	1.6	0.26
Immune induced molecule 1	IM1	3.4	0.00	1.6	0.17
Drosomycin	Drs	4.5	0.00	1.2	0.63
Peptidoglycan-recognition protein SD	PGRP-SD	3.7	0.00	1.4	0.15
Peptidoglycan recognition protein LF	PGRP-LF	1.6	0.03	-1.4	0.14
Melanization Protease 1	MP1	2.1	0.00	1.0	0.81
Relish	Rel	2.3	0.01	-1.4	0.26
Peptidoglycan recognition protein LB	PGRP-LB	1.9	0.02	1.1	0.59
Toll	Tl	1.8	0.00	-1.3	0.10
pelle	pll	1.6	0.00	1.0	0.84
immune deficiency	imd	1.6	0.03	-1.4	0.14
Serpin-27A	Spn27A	1.8	0.03	-1.1	0.78
Immune induced molecule 2	IM2	1.9	0.01	1.0	1.00
Immune induced molecule 4	IM4	4.0	0.00	1.7	0.15
Immune induced molecule 18; See Flybase.org FBgn0260455 and FBgn0067903.	---	1.6	0.02	1.1	0.56

Serine-type Peptidase Activity (IEF only)

**Title**	**Symbol**	**EF**	**P**	**SF**	**P**
Serine Protease Immune Response Integrator	spirit	3.1	0.00	1.2	0.31
Serine Protease 2	Ser7	4.5	0.00	1.7	0.03
Dmel_CG8952	CG8952	2.9	0.01	1.3	0.49
Dmel_CG6361	CG6361	2.6	0.00	1.2	0.27
Dmel_CG15046	CG15046	1.6	0.01	-1.0	0.96
Dmel_CG11911	CG11911	21.9	0.00	5.1	0.00
Dmel_CG3117	CG3117	1.7	0.00	-1.7	0.00
Dmel_CG11034	CG11034	3.5	0.00	1.3	0.20
Dmel_CG18563	CG18563	19.6	0.00	-1.1	0.69
Dmel_CG8299	CG8299	3.9	0.00	1.7	0.08
Dmel_CG10764	CG10764	8.5	0.00	1.2	0.23
Jonah 65Ai	Jon65Ai	12.6	0.00	5.5	0.03
Dmel_CG18179	CG18179	6.4	0.00	2.3	0.14
Dmel_CG10663	CG10663	1.6	0.01	-1.2	0.28
Jonah 74E	Jon74E	5.5	0.01	2.3	0.12
ATPase synthase, subunit d /// ---	ATPsyn-d ///CG14642	1.6	0.04	-1.2	0.36
Melanization Protease 1	MP1	2.1	0.00	1.0	0.81
Dmel_CG9649	CG9649	2.3	0.00	-1.2	0.43
Dmel_CG31326	CG31326	2.9	0.00	1.2	0.30
Dmel_CG9631	CG9631	2.3	0.00	1.2	0.14
Dmel_CG5246	CG5246	5.6	0.00	2.3	0.07
Dmel_CG5909	CG5909	2.5	0.03	-1.1	0.86
Dmel_CG11842	CG11842	2.8	0.01	1.0	0.96
POAE-like Serine protease	CG9733	4.6	0.00	2.0	0.14
deltaTrypsin; gammaTrypsin	CG30031 /// CG4269	2.2	0.00	1.1	0.54
Dmel_CG18754	CG18754	4.6	0.00	-1.1	0.58
anon-SAGE:Wang-110	CG17242	11.6	0.00	5.9	0.03
Dmel_CG30083	CG30083	1.6	0.01	-1.1	0.59
Dmel_CG30087	CG30087	2.0	0.00	1.1	0.66
Dmel_CG30091	CG30091	34.1	0.00	1.4	0.08
Dmel_CG30098	CG30098	5.5	0.01	1.8	0.29
Serine-peptidase 212	Sp212	3.4	0.00	1.2	0.44
Dmel_CG33462	CG33462	43.9	0.00	-1.3	0.70
Dmel_CG34043	CG34043	3.7	0.02	1.3	0.60
Dmel_CG34295	CG34295	1.5	0.03	1.1	0.66

Response to Fungus (IEF only)

**Title**	**Symbol**	**EF**	**P**	**SF**	**P**
Serine Protease Immune Response Integrator	spirit	3.1	0.00	1.2	0.31
cactus	cact	2.0	0.02	-1.3	0.26
Metchnikowin	Mtk	10.3	0.00	1.5	0.55
drosomycin-2	dro2	2.0	0.03	-1.7	0.10
Drosomycin	Drs	4.5	0.00	1.2	0.63
Toll	Tl	1.8	0.00	-1.3	0.10
pelle	pll	1.6	0.00	1.0	0.84
Serpin-27A	Spn27A	1.8	0.03	-1.1	0.78
necrotic	nec	3.9	0.00	1.4	0.23

Toll Signaling (IEF only)

Title	Symbol	EF	P	SF	P
Peptidoglycan recognition protein SA	PGRP-SA	3.0	0.00	1.3	0.18
cactus	cact	2.0	0.02	-1.3	0.26
Immune induced molecule 10	CG33470 /// IM10	4.5	0.00	1.8	0.08
Immune induced molecule 23	IM23	4.7	0.00	1.6	0.26
Relish	Rel	2.3	0.01	-1.4	0.26
Toll	Tl	1.8	0.00	-1.3	0.10
pelle	pll	1.6	0.00	1.0	0.84
Serpin-27A	Spn27A	1.8	0.03	-1.1	0.78
necrotic	nec	3.9	0.00	1.4	0.23

Chitin Metabolism (overlap)

Title	Symbol	EF	P	SF	P
Imaginal disc growth factor 4	Idgf4	1.9	0.00	1.5	0.06
Dmel_CG34324	CG34324	1.6	0.11	1.8	0.04
Mucin related 18B	Mur18B	1.7	0.05	1.2	0.43
Dmel_CG8460	CG8460	1.5	0.00	1.3	0.00
Imaginal disc growth factor 2	Idgf2	2.1	0.00	1.6	0.00
Imaginal disc growth factor 3	Idgf3	1.6	0.02	1.4	0.11
Chitinase-like	CG5210	3.2	0.00	1.9	0.02
CG9357	Cht8	2.3	0.00	2.0	0.01
CG10531	Cht9	8.2	0.00	3.4	0.02
Dmel_CG13309	CG13309	2.8	0.05	2.8	0.05
Tequila	Tequila	1.8	0.01	1.3	0.22
Mucin 68D	Muc68D	1.7	0.03	2.3	0.00
Dmel_CG14125	CG14125	2.1	0.01	2.1	0.01
Hemolectin	Hml	1.6	0.10	2.0	0.02
Dmel_CG7017	CG7017	3.6	0.01	4.2	0.00
Dmel_CG14607	CG14607	1.8	0.00	1.5	0.00
Dmel_CG6403	CG6403	1.7	0.03	1.4	0.11
obstructor-I	obst-I	1.6	0.01	1.2	0.31
Dmel_CG33173	CG33173	3.1	0.03	3.5	0.02
Dmel_CG33258	CG33258	2.0	0.00	1.4	0.08
Dmel_CG34427	CG34427	2.7	0.00	1.8	0.03

Negative Regulation of Proteolysis (overlap)

Title	Symbol	EF	P	SF	P
Serpin 53F	Spn53F	1.7	0.00	1.3	0.07
Accessory gland peptide 62F	Acp62F	2.0	0.01	1.3	0.25
Accessory gland-specific peptide 76A	Acp76A	2.2	0.00	1.3	0.26
Serpin 77Bc	Spn77Bc	5.0	0.00	3.9	0.00
Serine protease inhibitor 4	Spn4	2.2	0.00	1.3	0.21
Serine protease inhibitor 3	Spn3	2.1	0.00	1.5	0.07
Serine protease inhibitor 2	Spn2	1.8	0.01	1.3	0.19

Hormone Metabolism (overlap)

Title	Symbol	EF	P	SF	P
phantom	phm	1.6	0.00	1.4	0.03
Probable cytochrome P450 4ac1	Cyp4ac1	1.3	0.29	1.9	0.02
Probable cytochrome P450 4ac2	Cyp4ac2	2.6	0.01	3.4	0.00
juvenile hormone acid methyltransferase	jhamt	2.2	0.00	1.3	0.25
Probable cytochrome P450 4aa1	Cyp4aa1	3.7	0.00	4.2	0.00
Juvenile hormone epoxide hydrolase 3	Jheh3	1.3	0.26	1.9	0.02
Esterase 6	Est-6	4.0	0.00	2.3	0.06
Serine protease inhibitor 4	Spn4	2.2	0.00	1.3	0.21
desaturase1	desat1	2.0	0.00	1.3	0.25
Juvenile hormone epoxide hydrolase 1	Jheh1	1.2	0.48	1.6	0.05

Defense Response (overlap)

Title	Symbol	EF	P	SF	P
lozenge	lz	1.6	0.00	1.3	0.04
CG9675	spheroide	1.7	0.01	1.4	0.08
Transferrin 1	Tsf1	2.9	0.00	2.2	0.01
Scavenger receptor class C, type I	Sr-CI	2.2	0.00	1.7	0.01
Turandot M	TotM	5.2	0.04	4.2	0.06
Dmel_CG9029	CG9029	1.9	0.01	1.3	0.32
Galactose-specific C-type lectin	Lectin-galC1	4.1	0.00	2.5	0.02
Thiolester containing protein IV	TepIV	3.5	0.00	1.9	0.03
Immune induced molecule 10	CG33470 /// IM10	4.5	0.00	1.8	0.08
Dmel_CG16799	CG16799	3.3	0.00	1.9	0.05
Dmel_CG2736	CG2736	2.1	0.01	2.5	0.00
drosomycin-5	dro5	3.0	0.00	2.0	0.02
CG32382	sphinx2	2.1	0.03	1.3	0.38
Sterile alpha and TIR motif-containing protein 1	Ect4	2.1	0.01	1.7	0.05
Gram-negative bacteria binding protein 3	GNBP3	1.3	0.17	1.8	0.01
Dmel_CG6168	CG6168	1.7	0.02	1.3	0.18
Hemolectin	Hml	1.6	0.10	2.0	0.02
Gram-negative bacteria binding protein 2	GNBP2	1.8	0.02	1.2	0.35
Spatzle-Processing Enzyme	SPE	2.7	0.00	1.4	0.12
spatzle	spz	1.6	0.04	1.2	0.52
Serine protease inhibitor 3	Spn3	2.1	0.00	1.5	0.07
Turandot X	TotX	4.5	0.03	3.7	0.05
Turandot C	TotC	6.2	0.06	12.0	0.02
CG32383	sphinx1	1.7	0.01	1.5	0.05

Response to Toxin (overlap)

Title	Symbol	EF	P	SF	P
phantom	phm	1.6	0.00	1.4	0.03
Probable serine hydrolase	kraken	1.6	0.02	1.5	0.05
Probable cytochrome P450 4ac1	Cyp4ac1	1.3	0.29	1.9	0.02
Probable cytochrome P450 4ac2	Cyp4ac2	2.6	0.01	3.4	0.00
Probable cytochrome P450 4aa1	Cyp4aa1	3.7	0.00	4.2	0.00
Juvenile hormone epoxide hydrolase 3	Jheh3	1.3	0.26	1.9	0.02
Probable cytochrome P450 304a1	Cyp304a1	2.1	0.04	2.1	0.03
Juvenile hormone epoxide hydrolase 1	Jheh1	1.2	0.48	1.6	0.05

Oxidation Reduction (ISF only)

Title	Symbol	EF	P	SF	P
Probable cytochrome P450 6t1	Cyp6t1	1.0	0.93	2.1	0.02
Dmel_CG6012	CG6012	-1.1	0.68	1.7	0.00
Probable cytochrome P450 6w1	Cyp6w1	1.1	0.69	1.8	0.05
Probable cytochrome P450 6a13	Cyp6a13	-1.0	0.82	1.5	0.03
Phenoloxidase subunit A3	PO45	1.2	0.36	5.2	0.00
Dmel_CG10131	CG10131	1.1	0.28	1.5	0.01
Black cells	proPO-A1	1.3	0.27	4.4	0.00
Pyrroline 5-carboyxlate reductase	P5cr	1.0	0.79	1.5	0.03

Notch Signaling (IEF only)

Title	Symbol	EF	P	SF	P
E(spl) region transcript m3	HLHm3	-1.5	0.04	-1.0	0.94
Presenilin	Psn	-1.5	0.02	-1.1	0.50
E(spl) region transcript mbeta	HLHmbeta	-1.5	0.02	-1.0	0.94

Epithelium Development (overlap)

Title	Symbol	EF	P	SF	P
G-protein coupled receptor moody	moody	-1.3	0.10	-1.5	0.01
Downstream of kinase	Dok	-1.9	0.00	-1.7	0.01
unconventional myosin class XV	Myo10A	-2.0	0.00	-2.0	0.00
discs large 1	dlg1	-1.6	0.01	-1.5	0.02
lethal (2) giant larvae	l(2)gl	-1.2	0.24	-1.6	0.01
dachsous	ds	-1.9	0.02	-1.7	0.04
anterior open	aop	-2.4	0.00	-2.3	0.00
echinoid	ed	-1.7	0.05	-1.9	0.02
fat	ft	-3.1	0.00	-3.3	0.00
Btk family kinase at 29A	Btk29A	-1.4	0.06	-1.6	0.01
dachs	d	-3.0	0.00	-2.8	0.00
escargot	esg	-1.3	0.05	-1.7	0.00
Gliotactin	Gli	-1.8	0.02	-2.1	0.01
Van Gogh	Vang	-1.3	0.13	-1.5	0.03
scab	scb	-1.6	0.11	-1.9	0.02
grainy head	grh	-1.5	0.05	-1.4	0.08
four-jointed	fj	-3.3	0.00	-1.8	0.03
sprouty	sty	-1.2	0.39	-1.8	0.01
shade	shd	-1.6	0.00	-1.2	0.07
Protein giant-lens	aos	-2.1	0.00	-2.1	0.00
inturned	in	-2.5	0.00	-2.5	0.00
yurt	yrt	-1.3	0.00	-1.5	0.00
Stubble	Sb	-1.4	0.06	-1.5	0.02
Delta	Dl	-2.4	0.00	-2.5	0.00
branchless	bnl	-1.4	0.09	-1.7	0.02
Rho-kinase	rok	-1.3	0.15	-2.2	0.00
pericardin	prc	-5.2	0.00	-3.7	0.02
PAK-kinase	Pak	-1.2	0.28	-1.5	0.02
Frizzled	fz	-1.7	0.01	-1.6	0.02
krotzkopf verkehrt	kkv	-5.1	0.00	-4.8	0.00
knickkopf	knk	-4.0	0.00	-3.1	0.00
Myosin binding subunit	Mbs	-1.3	0.18	-1.5	0.03

Polysaccharide Metabolism (overlap)

Title	Symbol	EF	P	SF	P
CG2989	Cht6	-2.7	0.05	-3.2	0.03
obstructor-A	obst-A	-2.6	0.00	-3.3	0.00
Peritrophin A	Peritrophin-A	-2.5	0.00	-2.5	0.00
CG32499	Cda4	-3.1	0.00	-2.0	0.01
CG31973	Cda5	-2.6	0.00	-3.7	0.00
obstructor-B	obst-B	-3.2	0.00	-2.0	0.01
Dmel_CG8192	CG8192	-2.0	0.02	-1.8	0.05
Chitinase 2	Cht2	-1.7	0.02	-1.5	0.09
CG1869	Cht7	-2.7	0.00	-3.1	0.00
cracked	ckd	-1.5	0.01	-2.0	0.00
Dmel_CG4835	CG4835	-1.5	0.06	-2.0	0.00
Dmel_CG13676	CG13676	-2.5	0.00	-2.9	0.00
Dmel_CG32036	CG32036	-1.5	0.00	-1.3	0.03
Dmel_CG11570	CG11570	-1.2	0.28	-1.5	0.01
LDLa domain containing chitin binding protein 1	verm	-2.6	0.01	-3.8	0.00
serpentine	serp	-2.2	0.01	-2.6	0.00
Gasp	Gasp	-2.9	0.00	-3.0	0.00
chitin-binding peritrophin-A	Cht5	-11.1	0.00	-11.2	0.00
Dmel_CG14304	CG14304	-1.8	0.00	-1.9	0.00
Dmel_CG14301	CG14301	-1.6	0.03	-1.9	0.01

Dmel_CG7714	CG7714	-2.4	0.00	-2.9	0.00
krotzkopf verkehrt	kkv	-5.1	0.00	-4.8	0.00
knickkopf	knk	-4.0	0.00	-3.1	0.00
Dmel_CG13643	CG13643	-3.3	0.00	-2.9	0.00

Appendage Morphogenesis (overlap)

Title	Symbol	EF	P	SF	P
Downstream of kinase	Dok	-1.9	0.00	-1.7	0.01
furrowed	fw	-1.6	0.02	-1.8	0.01
forked	f	-2.3	0.00	-2.1	0.01
dachsous	ds	-1.9	0.02	-1.7	0.04
echinoid	ed	-1.7	0.05	-1.9	0.02
fat	ft	-3.1	0.00	-3.3	0.00
dachs	d	-3.0	0.00	-2.8	0.00
Gliotactin	Gli	-1.8	0.02	-2.1	0.01
dachshund	dac	-1.5	0.04	-1.4	0.11
shavenoid	sha	-1.8	0.01	-1.2	0.35
downstream of receptor kinase	drk	-1.2	0.32	-1.5	0.04
four-jointed	fj	-3.3	0.00	-1.8	0.03
wing morphogenesis defect	wmd	-1.6	0.09	-1.9	0.03
vein	vn	-1.5	0.06	-1.7	0.03
Protein giant-lens	aos	-2.1	0.00	-2.1	0.00
Wrinkled	W	-3.8	0.01	-2.4	0.05
tricornered	trc	-1.6	0.00	-1.5	0.00
inturned	in	-2.5	0.00	-2.5	0.00
fringe	fng	-1.5	0.01	-1.7	0.00
homothorax	hth	-1.5	0.02	-1.8	0.00
Delta	Dl	-2.4	0.00	-2.5	0.00
bursicon	burs	-1.9	0.03	-2.7	0.00
held out wings	how	-1.6	0.01	-1.5	0.02
Rho-kinase	rok	-1.3	0.15	-2.2	0.00
split ends	spen	-1.5	0.05	-1.3	0.20
miniature	m	-1.8	0.00	-1.6	0.00
Frizzled	fz	-1.7	0.01	-1.6	0.02
net	net	-1.8	0.01	-1.6	0.02
Myosin binding subunit	Mbs	-1.3	0.18	-1.5	0.03

Regulation of Neurogenesis (overlap)

Title	Symbol	EF	P	SF	P
bifocal	bif	-1.6	0.02	-1.6	0.02
Rho GTPase-activating protein 190	RhoGAPp190	-1.3	0.10	-1.5	0.02
lethal (2) giant larvae	l(2)gl	-1.2	0.24	-1.6	0.01
anterior open	aop	-2.4	0.00	-2.3	0.00
echinoid	ed	-1.7	0.05	-1.9	0.02
brain tumor	brat	-2.9	0.00	-3.2	0.00
Fragile X mental retardation syndrome-related protein 1	Fmr1	-1.5	0.01	-1.6	0.00
sprouty	sty	-1.2	0.39	-1.8	0.01
seven in absentia	sina	-1.5	0.05	-1.4	0.11
tricornered	trc	-1.6	0.00	-1.5	0.00
Delta	Dl	-2.4	0.00	-2.5	0.00
still life	sif	-1.2	0.13	-1.6	0.00
Rho-kinase	rok	-1.3	0.15	-2.2	0.00
PAK-kinase	Pak	-1.2	0.28	-1.5	0.02
Myosin binding subunit	Mbs	-1.3	0.18	-1.5	0.03

Cell Adhesion (overlap)

Title	Symbol	EF	P	SF	P
roughest	rst	-2.1	0.00	-1.8	0.00
discs large 1	dlg1	-1.6	0.01	-1.5	0.02
furrowed	fw	-1.6	0.02	-1.8	0.01
dachsous	ds	-1.9	0.02	-1.7	0.04
echinoid	ed	-1.7	0.05	-1.9	0.02
fat	ft	-3.1	0.00	-3.3	0.00
SP1070	uif	-2.2	0.00	-2.5	0.00
Dmel_CG7227	CG7227	-2.2	0.00	-1.8	0.01
Fasciclin 3	Fas3	-1.5	0.00	-1.7	0.00
scab	scb	-1.6	0.11	-1.9	0.02
lysyl oxidase-like 2	lox2	-2.9	0.00	-1.7	0.01
Matrix metalloproteinase 1	Mmp1	-2.2	0.06	-2.9	0.02
Ninjurin A	NijA	-1.5	0.10	-2.7	0.00
Cad74A	Cad74A	-1.9	0.01	-2.2	0.00
neuromusculin	nrm	-1.3	0.14	-1.7	0.00
Amalgam	Ama	-2.8	0.00	-2.7	0.00
Cadherin-87A	Cad87A	-1.7	0.00	-1.7	0.00
Ninjurin C	CG14394	-2.5	0.00	-2.4	0.00
Delta	Dl	-2.4	0.00	-2.5	0.00
held out wings	how	-1.6	0.01	-1.5	0.02
Cad99C	Cad99C	-1.8	0.02	-1.9	0.01
Pten	Pten	-1.5	0.10	-1.6	0.05
PAK-kinase	Pak	-1.2	0.28	-1.5	0.02
Frizzled	fz	-1.7	0.01	-1.6	0.02

Gamete Generation (ISF only)

Title	Symbol	EF	P	SF	P
strawberry notch	sno	-1.1	0.48	-1.6	0.01
Bicaudal D	BicD	1.1	0.63	-1.6	0.00
wunen	wun	1.2	0.36	-1.7	0.01
Myocyte enhancing factor 2	Mef2	1.0	0.96	-1.5	0.02
tout-velu	ttv	-1.0	0.81	-1.5	0.03
Ras-related protein Rac1	Rac1	-1.1	0.62	-1.6	0.01
Protein tyrosine phosphatase 61F	Ptp61F	1.0	0.86	-1.5	0.00
rhomboid	rho	-1.1	0.73	-1.6	0.01
Ecdysone-induced protein 75B	Eip75B	-1.0	0.95	-1.7	0.02
cAMP-dependent protein kinase R1	Pka-R1	-1.0	0.85	-1.5	0.04
puckered	puc	-1.1	0.52	-1.6	0.02
PI3 kinase	Pi3K92E	-1.1	0.15	-1.7	0.00
Axin	Axn	-1.0	0.90	-1.5	0.02
misshapen	msn	-1.1	0.57	-1.7	0.01
encore	enc	-1.1	0.66	-1.5	0.02
spire	spir	1.0	0.97	-1.5	0.02
tramtrack	ttk	-1.1	0.29	-1.6	0.00
G protein-coupled receptor kinase 2	Gprk2	1.1	0.67	-1.6	0.03
Basigin	Bsg	-1.1	0.33	-1.8	0.00
Sex lethal	Sxl	-1.1	0.68	-1.6	0.05

Phosphate Metabolism (ISF only)

Title	Symbol	EF	P	SF	P
Furin 2	Fur2	-1.1	0.63	-1.7	0.03
PRL-1	PRL-1	-1.0	0.78	-1.5	0.00
Dmel_CG7180	CG7180	1.0	0.89	-1.5	0.01
Src oncogene at 42A	Src42A	-1.0	0.75	-1.5	0.02
wunen	wun	1.2	0.36	-1.7	0.01
Stretchin-Mlck	Strn-Mlck	-1.0	0.87	-1.6	0.02
Protein tyrosine phosphatase 61F	Ptp61F	1.0	0.86	-1.5	0.00
Tie-like receptor tyrosine kinase	Tie	-1.0	0.91	-1.8	0.02
puckered	puc	-1.1	0.52	-1.6	0.02
Vacuolar H[+]-ATPase 55kD B subunit	Vha55	-1.0	0.91	-1.5	0.03
center divider	cdi	1.7	0.00	-1.5	0.02
PI3 kinase	Pi3K92E	-1.1	0.15	-1.7	0.00
Protein phosphatase 1alpha at 96A	Pp1alpha-96A	-1.1	0.51	-1.5	0.04
Protein C kinase 98E	Pkc98E	-1.1	0.47	-1.6	0.00
misshapen	msn	-1.1	0.57	-1.7	0.01
G protein-coupled receptor kinase 2	Gprk2	1.1	0.67	-1.6	0.03
Tousled-like kinase	tlk	-1.1	0.66	-1.6	0.01

Cell Motion (ISF only)

Title	Symbol	EF	P	SF	P
Troponin C isoform 4	TpnC4	1.1	0.77	-1.5	0.05
Src oncogene at 42A	Src42A	-1.0	0.75	-1.5	0.02
wunen	wun	1.2	0.36	-1.7	0.01
tout-velu	ttv	-1.0	0.81	-1.5	0.03
Semaphorin-5c	Sema-5c	-1.1	0.61	-1.5	0.01
Ras-related protein Rac1	Rac1	-1.1	0.62	-1.6	0.01
Protein tyrosine phosphatase 61F	Ptp61F	1.0	0.86	-1.5	0.00
knockout	ko	-1.1	0.58	-1.7	0.02
puckered	puc	-1.1	0.52	-1.6	0.02
mical	Mical	-1.1	0.74	-1.6	0.04
branchless	bnl	-1.0	0.86	-1.6	0.01
lethal (3) s2172	cp309	1.1	0.48	-1.6	0.02
