# Supplementary material for: Toll Mediated Infection Response Is Altered by Gravity and Spaceflight in Drosophila
Source: PLoS One. 2014 Jan 24;9(1):e86485. doi: 10.1371/journal.pone.0086485 (PMC3901686; doi:10.1371/journal.pone.0086485)
Supplement: Table S2 — Individual genes for all categories of the response to E. coli in Figure 5. (PDF) [file pone.0086485.s002.pdf]

Table S2. Individual genes (Title and Symbol) for all categories of the response to E. coli in Figure 5. Fold change in earth flies (EB), space flies (SB), and corresponding P values (P).

Transcription Regulation (IEB only)

Title	Symbol	EB	P	SB	P
nubbin	nub	1.7	0.01	-1.4	0.05
Cyclic-AMP response element binding protein A	CrebA	1.9	0.01	1.1	0.52
Sex comb on midleg	Fst // Scm	12.4	0.00	2.8	0.09
seven up	svp	1.6	0.02	1.0	0.99
forkhead box, sub-group O	foxo	1.6	0.04	1.1	0.59
fruitless	fru	1.5	0.00	1.1	0.54
Signal-transducer and activator of transcription protein at 92E	Stat92E	1.5	0.03	1.1	0.45
Trithorax-like	Trl	1.5	0.03	-1.0	0.85
kayak	kay	1.7	0.00	1.1	0.45

Cell Morphogenesis (IEB only)

Title	Symbol	EB	P	SB	P
Mps one binder kinase activator-like 2	Mob2	1.6	0.01	1.0	0.76
p130CAS	p130CAS	1.5	0.03	1.1	0.62
rhomboid	rho	1.6	0.02	-1.4	0.09
Sex comb on midleg	Fst /// Scm	12.4	0.00	2.8	0.09
seven up	svp	1.6	0.02	1.0	0.99
Signal-transducer and activator of transcription protein at 92E	Stat92E	1.5	0.03	1.1	0.45
kayak	kay	1.7	0.00	1.1	0.45

Innate Immunity (overlap)

Title	Symbol	EB	P	SB	P
Peptidoglycan recognition protein SA	PGRP-SA	6.2	0.00	5.0	0.00
Turandot M	TotM	20.6	0.00	27.9	0.00
Peptidoglycan-recognition protein SC2	PGRP-SC2	15.3	0.00	8.9	0.00
Defensin	Def	186.4	0.00	156.1	0.00
Attacin-C	AttC	14.9	0.00	15.7	0.00
Immune induced molecule 10	CG33470	3.7	0.00	2.2	0.04
Drosocin	Dro	68.3	0.00	77.8	0.00
Attacin-A	AttA	82.2	0.00	68.4	0.00
Metchnikowin	Mtk	26.2	0.00	32.4	0.00
Immune induced molecule 23	IM23	3.8	0.01	3.1	0.02
Immune induced molecule 1	IM1	3.4	0.00	3.1	0.00
Peptidoglycan-recognition protein SD	PGRP-SD	9.8	0.00	8.4	0.00
Sterile alpha and TIR motif-containing protein 1	Ect4	1.7	0.06	2.0	0.02
Peptidoglycan recognition protein LA	PGRP-LA	2.1	0.01	2.2	0.01
Peptidoglycan recognition protein LC	PGRP-LC	2.4	0.00	2.0	0.00
Peptidoglycan recognition protein LF	PGRP-LF	5.6	0.00	3.8	0.00
TGF-beta activated kinase 1	Tak1	1.6	0.00	1.4	0.01
Peptidoglycan-recognition protein SB1	PGRP-SB1	10.0	0.00	13.1	0.00
Relish	Rel	3.8	0.00	2.2	0.02
Peptidoglycan recognition protein LB	PGRP-LB	8.6	0.00	8.9	0.00
Cecropin B	CecB	4.9	0.00	5.4	0.00
Cecropin C	CecC	24.2	0.00	30.9	0.00
Turandot A	TotA	5.5	0.02	6.8	0.01
Immune induced molecule 2	IM2	2.0	0.00	1.8	0.01
Immune induced molecule 4	IM4	3.1	0.01	2.9	0.01
Turandot X	TotX	2.2	0.21	3.8	0.04
Turandot C	TotC	28.0	0.00	58.2	0.00
Immune induced molecule 18	---	1.9	0.00	2.1	0.00

Protein Biosynthesis (overlap)

Title	Symbol	EB	P	SB	P
Eukaryotic translation initiation factor 3 subunit G-1	CG8636	1.3	0.23	1.6	0.03
Probable phenylalanyl-tRNA synthetase alpha chain	CG2263	1.5	0.01	1.8	0.00
Lysyl-tRNA synthetase	Aats-lys	1.3	0.22	1.6	0.03
Eukaryotic translation initiation factor 2 subunit 1	eIF-2alpha	1.8	0.03	2.4	0.00
Ribosomal protein LP1	RpLP1	1.3	0.07	1.5	0.02
Ribosomal protein L40	RpL40	1.3	0.24	1.6	0.03
Eukaryotic translation initiation factor 3 subunit I	Trip1	1.3	0.07	1.7	0.00
Ribosomal protein S13	RpS13	1.6	0.06	1.7	0.05
string of pearls	RpS2	1.4	0.07	2.1	0.00
Asparaginyl-tRNA synthetase	Aats-asn	1.3	0.02	1.6	0.00
Eukaryotic translation initiation factor 3 subunit J	Adam	1.3	0.16	1.5	0.04
Phenylalanyl-tRNA synthetase	Aats-phe	1.4	0.08	1.9	0.00
Transport and Golgi organization 7	Tango7	1.6	0.05	1.7	0.02
Ribosomal protein LP2	RpLP2	1.5	0.03	1.5	0.03
Eukaryotic translation initiation factor 3 subunit B	eIF3-S9	1.6	0.02	1.5	0.04
Eukaryotic translation initiation factor 3 subunit K	CG10306	1.3	0.09	1.5	0.03
Probable eukaryotic translation initiation factor 6	eIF6	1.3	0.23	2.0	0.01
eIF4E-4	eIF4E-4	1.4	0.03	1.6	0.01
eIF4E-5	eIF4E-5	1.4	0.02	1.5	0.01
Eukaryotic initiation factor 2beta	eIF-2beta	1.4	0.03	2.0	0.00
Ribosomal protein S12	RpS12	1.4	0.04	1.6	0.01
Int6 homologue	Int6	1.4	0.05	1.7	0.00
eukaryotic release factor 1	eRF1	1.7	0.01	1.7	0.02

Eukaryotic translation initiation factor 2A	CG7414	1.6	0.17	2.2	0.02
Suppressor of variegation 3-9	Su(var)3-9	2.0	0.00	2.3	0.00
Elongation Factor G2	EF-G2	1.5	0.07	1.7	0.02
Eukaryotic initiation factor 3 p66 subunit	eIF-3p66	1.4	0.15	1.9	0.02
Glutamyl-prolyl-tRNA synthetase	Aats-glupro	1.4	0.02	1.9	0.00
Probable phenylalanyl-tRNA synthetase beta chain	CG5706	1.2	0.11	1.5	0.00
Ribosomal protein L4	RpL4	1.4	0.18	1.8	0.04
Tryptophanyl-tRNA synthetase	Aats-trp	1.5	0.04	1.7	0.01
Valyl-tRNA synthetase	Aats-val	1.8	0.00	2.3	0.00
Isoleucyl-tRNA synthetase	Aats-ile	1.5	0.01	1.5	0.01
Glutaminyl-tRNA synthetase	Aats-gln	1.3	0.11	1.8	0.00
4EHP	4EHP	1.7	0.13	2.0	0.04

Response to Bacterium (overlap)

Title	Symbol	EB	P	SB	P
Serine Protease Immune Response Integrator	spirit	6.8	0.00	6.4	0.00
Peptidoglycan recognition protein SA	PGRP-SA	6.2	0.00	5.0	0.00
insulin-stimulated eIF-4E binding protein	Thor	2.5	0.02	2.2	0.04
Thiolester containing protein II	TepII	5.2	0.00	4.0	0.00
Thiolester containing protein I	TepI	22.2	0.00	28.4	0.00
Galactose-specific C-type lectin	Lectin-galC1	2.5	0.02	1.4	0.37
Thiolester containing protein IV	TepIV	2.7	0.00	2.9	0.00
Defensin	Def	186.4	0.00	156.1	0.00
eiger	egr	2.4	0.05	1.6	0.28
Attacin-C	AttC	14.9	0.00	15.7	0.00
Immune induced molecule 10	CG33470 // IM10	3.5	0.00	2.8	0.01
Drosocin	Dro	68.3	0.00	77.8	0.00
Metchnikowin	Mtk	26.2	0.00	32.4	0.00
Inhibitor of apoptosis 2	Iap2	1.8	0.02	1.9	0.01
Immune induced molecule 23	IM23	3.8	0.01	3.1	0.02
Diptericin B	DptB	26.6	0.00	40.1	0.00
CG15678	pirk	5.8	0.00	3.8	0.00
Drosomycin	Drs	5.5	0.00	5.4	0.00
Peptidoglycan-recognition protein SD	PGRP-SD	9.8	0.00	8.4	0.00
Peptidoglycan recognition protein LA	PGRP-LA	2.1	0.01	2.2	0.01
Peptidoglycan recognition protein LC	PGRP-LC	2.4	0.00	2.0	0.00
Relish	Rel	3.8	0.00	2.2	0.02
Peptidoglycan recognition protein LB	PGRP-LB	8.6	0.00	8.9	0.00
Attacin-D	AttD	154.1	0.00	99.5	0.00
Spatzle-Processing Enzyme	SPE	1.6	0.04	2.2	0.00
spatzle	spz	3.1	0.00	2.7	0.00
Gram-positive Specific Serine protease	grass	1.9	0.04	2.2	0.02
Cecropin B	CecB	4.9	0.00	5.4	0.00
Cecropin C	CecC	24.2	0.00	30.9	0.00
Turandot A	TotA	5.5	0.02	6.8	0.01
Turandot X	TotX	2.2	0.21	3.8	0.04

Humoral Immune Response (overlap)

Title	Symbol	EB	P	SB	P
insulin-stimulated eIF-4E binding protein	Thor	2.5	0.02	2.2	0.04
Thiolester containing protein II	TepII	5.2	0.00	4.0	0.00
Thiolester containing protein I	TepI	22.2	0.00	28.4	0.00
Galactose-specific C-type lectin	Lectin-galC1	2.5	0.02	1.4	0.37
Thiolester containing protein IV	TepIV	2.7	0.00	2.9	0.00
Defensin	Def	186.4	0.00	156.1	0.00
Attacin-C	AttC	14.9	0.00	15.7	0.00
Immune induced molecule 10	CG33470 // IM10	3.5	0.00	2.8	0.01
Drosocin	Dro	68.3	0.00	77.8	0.00
Metchnikowin	Mtk	26.2	0.00	32.4	0.00
Inhibitor of apoptosis 2	Iap2	1.8	0.02	1.9	0.01
Immune induced molecule 23	IM23	3.8	0.01	3.1	0.02
Diptericin B	DptB	26.6	0.00	40.1	0.00
Drosomycin	Drs	5.5	0.00	5.4	0.00
Peptidoglycan recognition protein LC	PGRP-LC	2.4	0.00	2.0	0.00
Hemolectin	Hml	3.3	0.00	2.6	0.00
ATP-dependent RNA helicase p62	Rm62	1.6	0.00	1.5	0.01
Relish	Rel	3.8	0.00	2.2	0.02
Attacin-D	AttD	154.1	0.00	99.5	0.00
spatzle	spz	3.1	0.00	2.7	0.00
Cecropin B	CecB	4.9	0.00	5.4	0.00
Cecropin C	CecC	24.2	0.00	30.9	0.00
longitudinals lacking	lola	1.9	0.01	2.0	0.00
necrotic	nec	1.5	0.18	2.0	0.03

Spindle Elongation (overlap)

Title	Symbol	EB	P	SB	P
stubarista	sta	1.4	0.19	1.7	0.03
Eukaryotic translation initiation factor 3 subunit G-1	CG8636	1.3	0.23	1.6	0.03
Ribosomal protein L17	RpL17	1.2	0.19	1.5	0.03
Eukaryotic translation initiation factor 2 subunit 1	eIF-2alpha	1.8	0.03	2.4	0.00
Ribosomal protein L27A	RpL27A	1.6	0.03	1.8	0.01
Ribosomal protein L36A	CG14645 // RpL36A	2.0	0.02	2.0	0.02
Ribosomal protein S13	RpS13	1.6	0.06	1.7	0.05
Ribosomal protein L21	RpL21	1.5	0.03	1.6	0.01
Ribosomal protein L31	RpL31	1.4	0.14	1.6	0.04

Ribosomal protein S15Ab	RpS15Aa // RpS15Ab	1.5	0.02	1.5	0.03
Ribosomal protein S15	RpS15	1.3	0.30	1.7	0.04
Ribosomal protein L11	RpL11	1.4	0.18	1.9	0.02
Ribosomal protein L39	RpL39	1.8	0.05	1.6	0.10
Ribosomal protein L19	RpL19	1.4	0.11	1.6	0.04
Ribosomal protein L28	RpL28	1.3	0.11	1.5	0.01
Ribosomal protein L18	RpL18	1.4	0.12	1.6	0.03
Ribosomal protein L14	RpL14	1.6	0.10	1.8	0.05
Ribosomal protein S9	RpS9	1.4	0.10	1.5	0.04
Eukaryotic initiation factor 2beta	eIF-2beta	1.4	0.03	2.0	0.00
Ribosomal protein L26	RpL26	1.7	0.08	1.8	0.05
Ribosomal protein S30	RpS30	1.3	0.38	1.8	0.04
eukaryotic translation initiation factor 4G	eIF4G	1.6	0.01	1.3	0.08
Ribosomal protein L37A	RpL37A	1.5	0.02	1.3	0.11
Ribosomal protein L12	RpL12	1.4	0.08	1.7	0.01

DNA Replication (ISB only)

Title	Symbol	EB	P	SB	P
Ribonucleoside diphosphate reductase small subunit	RnrS	1.1	0.61	1.6	0.01
mutagen-sensitive 209	mus209	1.0	0.86	1.5	0.01
Replication-factor-C 40kD subunit	RfC4	1.1	0.77	1.8	0.01
mitochondrial single stranded DNA-binding protein	mtSSB	-1.1	0.70	1.5	0.05
DNA polymerase alpha 60kD	DNApol-alpha60	1.0	0.92	1.6	0.00
DNA polymerase gamma 35kD	DNApol-gamma35	1.1	0.54	1.5	0.00

Translation (ISB only)

Title	Symbol	EB	P	SB	P
eIF2B-beta	eIF2B-beta	1.0	0.77	1.5	0.00
eIF2B-gamma	eIF2B-gamma	1.1	0.68	1.5	0.00
mitochondrial ribosomal protein S28	mRpS28	-1.1	0.72	1.8	0.01
mitochondrial ribosomal protein L17	mRpL17	-1.0	0.93	1.8	0.05
mitochondrial ribosomal protein S35	mRpS35	1.1	0.82	1.9	0.04
mitochondrial ribosomal protein S6	mRpS6	1.1	0.55	1.5	0.05
mitochondrial ribosomal protein S26	mRpS26	-1.0	0.90	1.5	0.02
mitochondrial ribosomal protein L21	mRpL21	-1.1	0.54	1.5	0.01
Rlc1	Rlc1	1.0	0.82	1.5	0.01
mitochondrial ribosomal protein S22	mRpS22	-1.0	0.86	1.9	0.01
mitochondrial ribosomal protein L12	mRpL12	1.1	0.64	1.8	0.01
Eukaryotic initiation factor 3 p40 subunit	eIF-3p40	1.1	0.57	1.7	0.03
DNA polymerase gamma 35kD	DNApol-gamma35	1.1	0.54	1.5	0.00

Protein Complex Assembly (ISB only)

Title	Symbol	EB	P	SB	P
Mediator complex subunit 31	MED31	1.1	0.53	1.8	0.01
alpha-Tubulin at 85E	alphaTub85E	1.1	0.49	1.5	0.03
Mediator complex subunit 7	MED7	1.0	0.85	1.7	0.02
TBP-associated factor 12	Taf12	1.0	0.84	1.6	0.01
Dmel_CG7794	CG7794	1.1	0.60	1.5	0.05
Surfeit 6	Surf6	1.1	0.58	1.5	0.02

Chitin Metabolism (IEB only)

Title	Symbol	EB	P	SB	P
obstructor-B	obst-B	-1.6	0.04	1.4	0.13
Dmel_CG13675	CG13675	-2.1	0.05	-1.1	0.79
Dmel_CG14304	CG14304	-1.5	0.03	-1.1	0.52

Metamorphosis (overlap)

Title	Symbol	EB	P	SB	P
Downstream of kinase	Dok	-1.6	0.02	-2.4	0.00
forked	f	-2.0	0.01	-2.7	0.00
BarH2	B-H2	-1.4	0.27	-2.5	0.01
BarH1	B-H1	-1.4	0.04	-1.9	0.00
Heparan sulfate 3-O sulfotransferase-B	Hs3st-B	-1.3	0.06	-1.5	0.01
decapentaplegic	dpp	-1.2	0.26	-1.6	0.01
scavenger receptor acting in neural tissue and majority of rhodopsin is absent	santa-maria	-1.3	0.11	-1.5	0.01
dachs	d	-2.2	0.02	-2.0	0.04
Gliotactin	Gli	-1.9	0.02	-2.2	0.00
costa	cos	-1.3	0.11	-1.5	0.01
downstream of receptor kinase	drk	-1.2	0.29	-1.9	0.00
Death caspase-1	Dcp-1	-1.7	0.03	-1.3	0.23
split central complex	Ptpmeg	-1.3	0.21	-1.6	0.05
Nedd2-like caspase	Nc	-1.7	0.04	-1.4	0.16
Protein giant-lens	aos	-1.5	0.08	-1.6	0.04
Ecdysone-induced protein 75B	Eip75B	-2.7	0.00	-2.3	0.01
odd paired	opa	-1.3	0.15	-1.8	0.01
doublesex	dsx	-1.3	0.12	-1.7	0.01
Daughters against dpp	Dad	-1.5	0.01	-1.4	0.03
bursicon	burs	-1.6	0.10	-2.4	0.00
Pten	Pten	-1.5	0.07	-1.9	0.01
cut	ct	-1.3	0.08	-1.7	0.00
miniature	m	-1.7	0.00	-1.7	0.00
Frizzled	fz	-1.3	0.22	-1.7	0.01
knot	kn	-1.5	0.05	-2.2	0.00

net	net	-1.4	0.09	-1.7	0.01
Ecdysone-inducible gene E1	ImpE1	-1.2	0.32	-1.5	0.02

Transcription Regulation (overlap)

Title	Symbol	EB	P	SB	P
Histone deacetylase complex subunit SAP30 homolog	Sap30	-1.4	0.01	-1.5	0.00
anterior open	aop	-1.6	0.05	-2.0	0.01
abrupt	ab	-1.5	0.09	-2.0	0.01
Hormone receptor-like in 38	Hr38	-1.7	0.06	-4.8	0.00
Hormone receptor-like in 39	Hr39	-1.4	0.04	-1.8	0.00
goliath	gol	-1.5	0.15	-2.2	0.01
trachealess	trh	-1.3	0.16	-1.6	0.02
estrogen-related receptor	ERR	-1.3	0.07	-1.6	0.00
Ecdysone-induced protein 75B	Eip75B	-2.7	0.00	-2.3	0.01
eagle	eg	-1.4	0.05	-1.5	0.04
odd paired	opa	-1.3	0.15	-1.8	0.01
Sex combs reduced	Scr	-1.2	0.27	-1.8	0.00
doublesex	dsx	-1.3	0.12	-1.7	0.01
single-minded	sim	-1.2	0.18	-1.5	0.01
Daughters against dpp	Dad	-1.5	0.01	-1.4	0.03
nautilus	nau	-1.6	0.05	-2.1	0.01
E(spl) region transcript m3	HLHm3	-1.7	0.02	-1.4	0.12
skuld	skd	-1.3	0.29	-1.7	0.03
cut	ct	-1.3	0.08	-1.7	0.00
knot	kn	-1.5	0.05	-2.2	0.00
tramtrack	ttk	-1.2	0.24	-1.5	0.01
modifier of mdg4	mod(mdg4) // pre-mod(mdg4)-AD //-AE //-W	-1.2	0.07	-1.6	0.00

Epithelium Development (overlap)

Title	Symbol	EB	P	SB	P
Ras-related protein	Rala	-1.2	0.46	-1.5	0.05
Downstream of kinase	Dok	-1.6	0.02	-2.4	0.00
unconventional myosin class XV	Myo10A	-1.9	0.00	-2.1	0.00
anterior open	aop	-1.6	0.05	-2.0	0.01
decapentaplegic	dpp	-1.2	0.26	-1.6	0.01
Btk family kinase at 29A	Btk29A	-1.3	0.16	-1.8	0.00
gurken	grk	-1.2	0.26	-1.5	0.02
dachs	d	-2.2	0.02	-2.0	0.04
Gliotactin	Gli	-1.9	0.02	-2.2	0.00
starry night	stan	-1.6	0.03	-1.4	0.17
shade	shd	-1.5	0.00	-1.6	0.00
Protein giant-lens	aos	-1.5	0.08	-1.6	0.04
Daughters against dpp	Dad	-1.5	0.01	-1.4	0.03
branchless	bnl	-1.3	0.15	-1.7	0.01
ADP-ribosylation factor-like 3	dnd	-2.0	0.06	-2.1	0.05
pericardin	prc	-2.9	0.05	-1.6	0.32
Frizzled	fz	-1.3	0.22	-1.7	0.01
knickkopf	knk	-2.2	0.01	-1.9	0.02

Programmed Cell Death (overlap)

Title	Symbol	EB	P	SB	P
roughest	rst	-1.3	0.07	-1.6	0.01
decapentaplegic	dpp	-1.2	0.26	-1.6	0.01
scavenger receptor acting in neural tissue and majority of rhodopsin is absent	santa-maria	-1.3	0.11	-1.5	0.01
Caspase-activated DNase	Drep-4	-1.6	0.05	-1.5	0.06
Rep3	Drep-3	-1.2	0.25	-1.6	0.03
Death caspase-1	Dcp-1	-1.7	0.03	-1.3	0.23
split central complex	Ptpmeg	-1.3	0.21	-1.6	0.05
Nedd2-like caspase	Nc	-1.7	0.04	-1.4	0.16
Ecdysone-induced protein 75B	Eip75B	-2.7	0.00	-2.3	0.01
sickle	skl	-1.8	0.01	-2.2	0.00
Pten	Pten	-1.5	0.07	-1.9	0.01
modifier of mdg4	mod(mdg4) // pre-mod(mdg4)-AD //-AE //-W	-1.2	0.07	-1.6	0.00
wunen-2	wun2	-1.3	0.25	-1.7	0.02

Actin Cytoskeleton Organization (ISB only)

Title	Symbol	EB	P	SB	P
G-protein coupled receptor moody	moody	-1.0	0.78	-1.5	0.03
LIM-kinase1	LIMK1	1.2	0.24	-1.5	0.02
inflated	if	-1.1	0.67	-1.7	0.01
flightless I	fliI	-1.1	0.52	-1.6	0.00
lethal (2) giant larvae	l(2)gl	1.1	0.44	-1.7	0.00
diaphanous	dia	1.1	0.53	-1.5	0.04
scrambled	sced	-1.0	0.75	-1.5	0.01
puckered	puc	-1.1	0.57	-2.4	0.00
Delta	Dl	-1.0	0.98	-1.9	0.01
Wiskott-Aldrich syndrome protein	WASp	1.1	0.50	-1.7	0.00

Ectoderm Development (ISB only)

Title	Symbol	EB	P	SB	P
LIM-kinase1	LIMK1	1.2	0.24	-1.5	0.02
Hormone receptor-like in 38	Hr38	-1.7	0.06	-4.8	0.00
puckered	puc	-1.1	0.57	-2.4	0.00
stripe	sr	-1.0	0.96	-1.9	0.00
Delta	Dl	-1.0	0.98	-1.9	0.01
hedgehog	hh	1.1	0.48	-1.7	0.01
Wiskott-Aldrich syndrome protein	WASp	1.1	0.50	-1.7	0.00

Cell Migration (ISB only)

Title	Symbol	EB	P	SB	P
small wing	sl	-1.1	0.45	-1.7	0.00
inflated	if	-1.1	0.67	-1.7	0.01
stathmin	stai	1.1	0.72	-1.5	0.03
escargot	esg	-1.1	0.56	-1.5	0.01
vein	vn	1.3	0.23	-1.6	0.05
puckered	puc	-1.1	0.57	-2.4	0.00
stripe	sr	-1.0	0.96	-1.9	0.00
Delta	Dl	-1.0	0.98	-1.9	0.01
held out wings	how	-1.1	0.66	-1.7	0.01
hedgehog	hh	1.1	0.48	-1.7	0.01
