# Supplementary material for: Toll Mediated Infection Response Is Altered by Gravity and Spaceflight in Drosophila
Source: PLoS One. 2014 Jan 24;9(1):e86485. doi: 10.1371/journal.pone.0086485 (PMC3901686; doi:10.1371/journal.pone.0086485)
Supplement: Table S3 — Genes with altered response in uninfected space flies, in addition to those in Table 2. (PDF) [file pone.0086485.s003.pdf]

Table S3. Genes with altered response in uninfected space flies, in addition to those in Table 2.

aay	(1.5),	Lcp65Ag3	(1.5),	sty	(1.6),	CG13311	(2.0),	CG31205	-(2.0),	CG5190	-(1.6),
Ast-CC	-(1.5),	Lip4	-(1.5),	sug	(1.8),	CG13321	(2.1),	CG31259	-(2.4),	CG5195	(1.9),
bchs	(1.6),	Lkr	(1.6),	Sur-8	(1.7),	CG13482	(3.1),	CG31272	(2.3),	CG5290	(2.3),
beat-IIIc	-(1.6),	Lpin	(1.7),	Taf7	(1.5),	CG13511	(2.6),	CG31288	-(1.6),	CG5348	(1.6),
bnl	(1.6),	Mob2	(1.7),	tamo	(1.6),	CG13604	(1.8),	CG31296	(1.6),	CG5550	(7.0),
Cda4	-(1.7),	MTF-1	(1.5),	Tdc1	(1.6),	CG13618	(2.0),	CG31337	(1.5),	CG5707	-(1.5),
cdi	(1.6),	MtnB	(2.8),	Tsp96F	(1.5),	CG13641	(1.8),	CG31370	(1.5),	CG6055	-(1.7),
cenG1A	(2.1),	nub	(1.9),	Uro	(2.4),	CG13773	-(1.5),	CG31626	(1.5),	CG6282	(1.5),
CHKov1	-(1.5),	Obp83g	-(1.7),	vimar	(1.5),	CG13833	(1.8),	CG31686	(2.2),	CG6767	(1.5),
CHMP2B	(1.8),	olf186-M	(1.5),	vir-1	(2.1),	CG14218	-(1.6),	CG31955	(1.8),	CG6910	(2.0),
Cht9	(2.8),	Osi12	(1.5),	Vrp1	(1.5),	CG14635	(1.6),	CG32107	(1.9),	CG6912	-(1.6),
Cyp12a4	(1.6),	osm-6	-(1.5),	WASp	(1.5),	CG14820	-(1.8),	CG32194	(1.7),	CG7031	-(1.7),
Cyp310a1	(1.7),	p38c	(2.1),	Whamy	(1.9),	CG14879	(1.5),	CG32553	(1.8),	CG7130	(1.8),
Cyp4p3	(1.6),	Pa1	(1.9),	yellow-e	-(1.5),	CG15024	-(1.5),	CG32564	-(1.5),	CG7135	(1.7),
Dhap-at	(1.7),	Pak	(1.5),	yellow-h	-(1.5),	CG15044	(2.2),	CG32850	(1.6),	CG7142	(2.9),
dia	(1.5),	pbl	(1.5),	ZnT35C	-(1.6),	CG15071	(1.6),	CG33474	(1.5),	CG7298	(3.2),
dl	(1.6),	Pde11	(1.7),	CG10051	-(2.2),	CG15366	(1.7),	CG3348	(1.7),	CG7458	-(1.5),
dpr18	(1.5),	PGRP-LF	(1.6),	CG10103	(1.7),	CG15745	(1.8),	CG33966	-(1.6),	CG7778	(1.6),
eIF2B-g	-(1.5),	pirk	(1.6),	CG10176	(1.5),	CG15817	(1.6),	CG34349	(1.6),	CG8005	-(1.5),
Eig71Ea	(1.5),	Pph13	(1.5),	CG10182	(4.2),	CG1673	(1.6),	CG34382	-(1.9),	CG8314	-(1.5),
fa2h	(1.6),	Pros45	-(1.5),	CG10300	(2.0),	CG17717	(1.5),	CG34396	(1.5),	CG8353	(1.5),
Fancl	(1.7),	puc	(2.0),	CG10337	(1.8),	CG17760	(1.6),	CG3760	-(1.5),	CG8679	(1.5),
Fst // Scm	(9.6),	Pvf1	(1.5),	CG10433	(2.4),	CG17786	-(1.5),	CG3764	(2.1),	CG8925	-(1.5),
GstD2	(4.0),	Rab9	(1.6),	CG10462	(1.6),	CG17930	(1.6),	CG3884	(2.2),	CG9084	(1.5),
GstD4	(2.7),	Rcd2	(1.8),	CG10476	-(1.5),	CG17974	-(1.7),	CG4168	(1.7),	CG9134	-(1.8),
GstD5	(2.1),	Rel	(2.4),	CG10514	-(1.9),	CG18446	(1.6),	CG42351	-(1.5),	CG9422	-(1.6),
GstE1	(1.9),	Rep	(1.5),	CG10516	-(1.8),	CG1850	(1.5),	CG42540	(1.6),	CG9452	(1.5),
GstE7	(2.7),	Rfx	(1.5),	CG10943	(1.9),	CG18563	(2.0),	CG42673	(1.6),	CG9568	(2.2),
GstE8//E9	(2.5),	rho-4	(1.5),	CG1129	(1.6),	CG18607	-(1.6),	CG42752	-(1.8),	CG9935	(1.5),
HLH54F	(1.5),	RhoGAP71E	(1.7),	CG11334	-(1.6),	CG18635	(1.7),	CG42817	(1.5),		
Hydr2	(1.5),	RpL3	(1.5),	CG11447	-(1.5),	CG18641	-(1.6),	CG4301	(1.5),		
Idgf1	(1.8),	RpLP0-like	-(1.5),	CG11825	(2.2),	CG18673	(1.9),	CG4313	-(1.7),		
inaF-B//F-D	(1.6),	SelD	-(1.5),	CG11889	(2.0),	CG18744	(2.7),	CG4576	(1.5),		
Ipk2	(1.8),	sha	-(1.5),	CG12187	(1.5),	CG1887	(1.5),	CG4702	-(1.8),		
jhamt	(1.8),	Spn28Db	(1.6),	CG12483	-(1.5),	CG30043	-(1.6),	CG4725	(1.6),		
lace	(1.5),	spz	(1.7),	CG12974	(1.5),	CG30160	(2.8),	CG4741	-(1.7),		
LamC	(1.8),	sr	(1.5),	CG13038	-(1.6),	CG30325	(1.5),	CG4753	-(2.0),		
lbk	(1.5),	Srp19	-(1.5),	CG13157	-(1.8),	CG31104	-(1.6),	CG4998	(2.4),		
